# Assembly processes and functional diversity of marine protists and their rare biosphere

**DOI:** 10.1186/s40793-023-00513-w

**Published:** 2023-07-13

**Authors:** Pierre Ramond, Raffaele Siano, Marc Sourisseau, Ramiro Logares

**Affiliations:** 1 Institute of Marine Sciences (ICM), Department of Marine Biology and Oceanography, CSIC, Barcelona, Catalunya 08003 Spain; 2 DYNECO/Pelagos, Ifremer-Centre de Brest, Technopôle Brest Iroise, Plouzané, 29280 France

**Keywords:** Community assembly, Rare biosphere, Marine protists, Community ecology, Functional ecology

## Abstract

**Background:**

The mechanisms shaping the rare microbial biosphere and its role in ecosystems remain unclear. We developed an approach to study ecological patterns in the rare biosphere and use it on a vast collection of marine microbiomes, sampled in coastal ecosystems at a regional scale. We study the assembly processes, and the ecological strategies constituting the rare protistan biosphere. Using the phylogeny and morpho-trophic traits of these protists, we also explore their functional potential.

**Results:**

Taxonomic community composition remained stable along rank abundance curves. *Conditionally rare taxa*, driven by selection processes, and *transiently rare taxa*, with stochastic distributions, were evidenced along the rank abundance curves of all size-fractions. Specific taxa within the divisions *Sagenista*, *Picozoa*, *Telonemia*, and *Choanoflagellida* were rare across time and space. The distribution of traits along rank abundance curves outlined a high functional redundancy between rare and abundant protists. Nevertheless, trophic traits illustrated an interplay between the trophic groups of different size-fractions.

**Conclusions:**

Our results suggest that rare and abundant protists are evolutionary closely related, most notably due to the high microdiversity found in the rare biosphere. We evidenced a succession of assembly processes and strategies of rarity along rank abundance curves that we hypothesize to be common to most microbiomes at the regional scale. Despite high functional redundancy in the rare protistan biosphere, *permanently rare protists* were evidenced, and they could play critical functions as bacterivores and decomposers from within the rare biosphere. Finally, changes in the composition of the rare protistan biosphere could be influenced by the trophic regime of aquatic ecosystems. Our work contributes to understanding the role of rare protists in microbiomes.

**Supplementary Information:**

The online version contains supplementary material available at 10.1186/s40793-023-00513-w.

## Background

A striking feature of environmental microbial communities is the dominance of few abundant taxa, which contrasts with a great diversity of rare ones [1, 2]. The existence of this “microbial rare biosphere” raised numerous questions over the past 20 years [1–4], many of which remain unanswered. A species can be rare spatially or temporally, rare locally or within a meta-community [5]. Studies about microbial rarity arose with the development of amplicon sequencing, which allowed to amplify and sequence marker genes to study community composition [6]. These first studies defined rarity based on arbitrary thresholds in species frequency, with e.g. abundant species representing > 1 or 0.1% and rare species representing < 0.1, 0.01 or 0.001% of the sequenced reads in a sample, or across a whole dataset [1, 2, 7]. Gradients in rarity have also been explored using Rank Abundance Curves (thereafter RACs) [8, 9], where species are attributed a rank and sorted based on their total number of reads in a community. Working with RACs computed at the meta-community level allows us to generalize results at the scale of the ecosystem(s) surveyed. Some authors have further argued that rare species are those species at the end of RACs from which the removal does not affect beta-diversity patterns [5, 10].

Rarity could represent a strategy to avoid competition, predation, parasitism, or viral infections [11], but the extent to which some phyla and taxa are rarer or more abundant is still unclear [1, 4, 12]. We also know that rare microbes obey assembly processes too [13], mainly: Selection (biotic and abiotic factors promoting or inhibiting the growth of taxa in an ecosystem), Dispersal (movement of taxa across an ecosystem), Diversification (generating new ecotypes, species, and lineages) and Ecological Drift (stochastic processes of birth and death that affect community composition) [14, 15]. Moreover, different strategies were evidenced as many taxa were shown to be *conditionally rare* and growing abundant under the right conditions [16–18], while some others were *transiently* (observed punctually and in low numbers) or *permanently rare* (found consistently in low numbers across the ecosystem surveyed) [7, 19]. A recent framework links types of rarity to the assembly processes acting upon them [5, 20]. In the following, we detail how different assembly processes shape different types of rarity within the rare biopshere. (1) *Variable selection* (changing through time and space) is thought to act upon *conditionally rare taxa*, which respond numerically depending on the biotic and abiotic conditions, in suitable conditions, these taxa might be able to bloom and escape the rare biosphere. (2) *Homogeneous selection* occurs when biotic and abiotic conditions stabilize community composition, taxa in the rare biosphere of these communities are thus *permanently rare*. (3) *Homogenizing dispersal* represents a physical input of neighboring populations, stable or periodic, that stabilizes community composition, it represents a second process that can explain the presence of *permanently rare taxa*. (4) Finally, *transiently rare taxa* could be influenced by both *dispersal limitation* and *stochastic processes* generating brief and random detection of some taxa from other ecosystems. Using this framework along RACs of microbial communities, we may be able to answer two main questions: (1) are there phyla unique to rarity and what are their ecological strategies? And (2) which assembly processes lead to the formation of the rare microbial biosphere?

The rare biosphere also questioned the link between microbial communities and ecosystem functioning [8]. Microbes drive ecosystems, prokaryotes are indeed involved in a wide array of metabolic pathways directly affecting biogeochemical cycles on Earth [21]. At the same time, protists produce, transform, and recycle organic matter, which fuels aquatic ecosystems [22]. Metabolic functions of the prokaryotes populating the rare biosphere can be investigated with metagenomics [6]. However, such approaches have yet to be widely used in protists. Still, approaches describing morphology and trophic behaviors could predict a protist’s functional role in ecosystems (e.g. phototroph, phagotroph, parasite, or detritivore) [23, 24]. An early hypothesis proposed that the rare microbial biosphere was functionally redundant [8], i.e., that rare microbes had the same ecosystem roles as their abundant counterparts. Indeed, perturbations were shown to induce blooms of *conditionally rare* taxa that carried out similar ecosystem functions [17, 18], suggesting that the rare biosphere could serve as a seed bank for ecosystem functions to be performed under disturbances or different environmental conditions [8]. Conversely, a recent hypothesis proposed that rare microbes are more likely to be functionally dissimilar from abundant ones, thus offering complementary or unique ecosystem functions, but also affecting the activity of the abundant taxa via their presence and/or secretions [3]. The existence of rare but active taxa [19, 25], some of them performing specific functions like soil sulfate reduction [26], or the degradation of particular compounds [2], supports this hypothesis. This debate raises a fundamental question: Do abundant and rare microbial taxa show contrasting ecological strategies and functional diversity, or are they functionally redundant?

In this study, we investigate pelagic protistan communities from several coastal ecosystems along the coast of France [24], and investigate the rare protistan biosphere at the meta-community scale across various connected marine habitats. With emphasis on three planktonic size-fractions (micro [> 20 or 10 μm], nano [20 or 10 − 3 μm], and pico-plankton [3-0.2 μm]), we investigate rarity based on rank-abundance-curves. We first compare the taxonomic composition of the rare biosphere to its abundant counterpart. We then investigate the assembly processes and ecological strategies shaping the rare protistan biosphere. Finally, using a previous annotation of morphological and trophic traits of the protistan taxa present in our dataset [24, 27], we question whether rare protist present different ecosystems roles.

## Materials and methods

### Environmental samples, amplicon sequencing and functional trait annotation

**Fig. 1:**
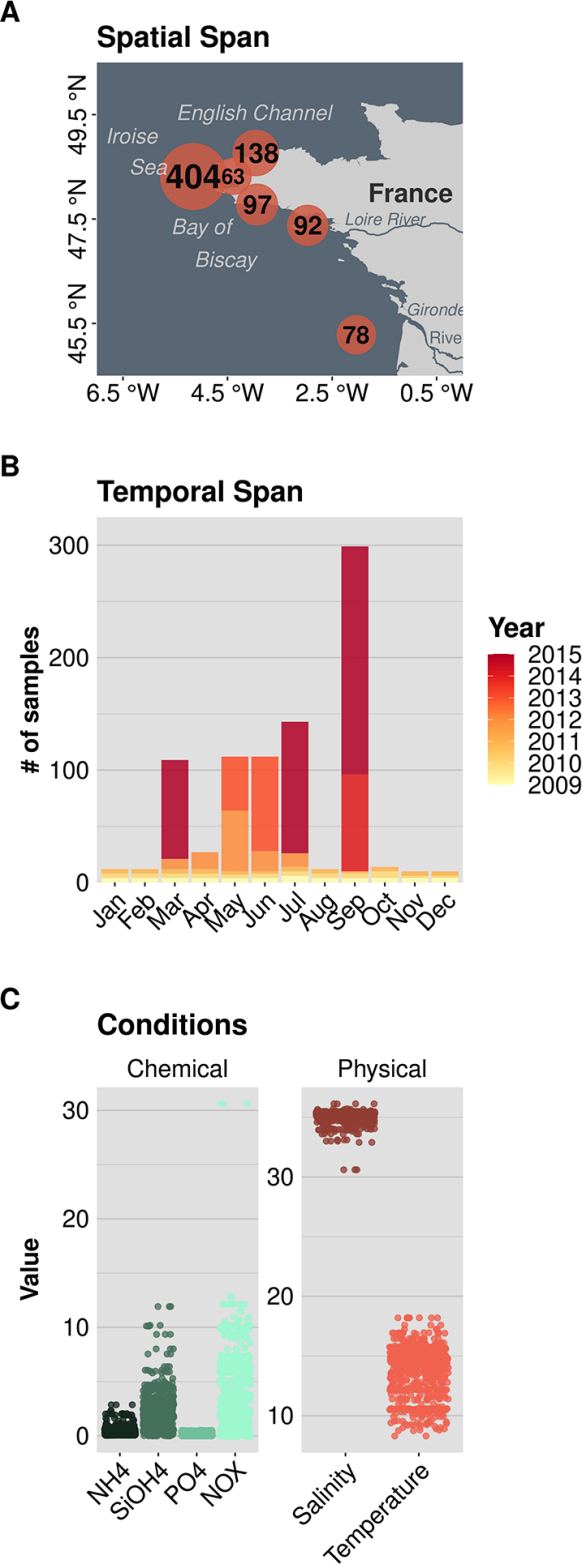
Ecological context of the samples from our dataset of marine protistan communities in North-Atlantic coastal Ecosystems (872 samples). (**A**) Spatial span of our survey. The center of the circles represents the spatial origin of the samples, the number of samples is displayed for each spatial region. (**B**) Temporal span of our survey (2009–2015). The barplots represent the number of samples across months and years (color code). (**C**) Environmental conditions sampled in our survey. Each circle represents the value of the corresponding variable in a sample. The units of nutrients, temperature and salinity are µM, PSU and °C

The samples of our dataset were collected along the French Atlantic coast (Fig. 1A). The collection spans the years 2009 to 2015, and most samples were taken between March and September (Fig. 1B), corresponding to spring and summer. Its spatial extent is relatively narrow (~ 4° in latitude and longitude, Fig. 1A) and covers mostly shallow ecosystems (average depth of 32 m across samples), where bottom and surface microbial communities can be mixed. The seawater in these ecosystems is known to harbor seasonal fronts affecting the regional connectivity between oceanic basins (e.g. between the English Channel and the Bay of Biscay) [28, 29], it is also influenced by the plumes of large estuaries (the Gironde and Loire rivers), with terrestrial, turbid, organically rich and hypoxic waters mixing with coastal and oceanic waters [30, 31], or by the seasonal cycle of physical and chemical conditions [32]. Sampling procedures and processing can be found in ref [24]. Briefly, coastal seawater was filtered sequentially on filters of pore size: 20 or 10 μm, 3 μm, and 0.2 μm to distinguish micro, nano, and pico-plankton, for a total of 1147 filters (367, 435, and 345 filters for micro, nano, and pico-plankton respectively). Parallel measurements were taken to characterize the physical (temperature, salinity) and chemical conditions (NO_3_^−^ + NO_2_^−^ = NOX, PO_4_^3−^, NH_4_^+^, and Si(OH)_4_) of the water masses sampled (Fig. 1C). Genomic DNA was extracted using the DNA extraction kit NucleoSpin Plant II (Macherey-Nagel, Hoerdt, France), and the V4 region of the 18 S rRNA gene was amplified and used as a universal eukaryotic marker gene, following [9]. Sequencing was performed at Genotoul (http://get.genotoul.fr/).

Bioinformatics are detailed in [24]. In summary, the quality-check was performed with USEARCH [33]. Clustering of reads into Operational Taxonomic Units (OTUs) was performed with Swarm2 (with a clustering threshold of d = 1) [34]; OTUs present in less than 2 samples and having less than 3 reads were removed as they could represent sequencing artefacts (following the ‘singleton removal’ of ref [35].). OTUs were taxonomically classified using the PR2 database [36] with an identity threshold of 80% (following ref [35].); Metazoans and multi-cellular plants were removed. From this original dataset, we excluded samples representing strong outliers in community patterns (datasets DA and SE in [24]). This subset consists of 257, 325, and 290 samples (872 in total); respectively for micro-, nano- and pico-plankton; and contains 90 432 OTUs and 2.26 × 10^7^ reads. Diversity saturation was investigated with R package ‘iNEXT’ [37], rarefaction curves, diversity estimation and complementary diversity analyses can be found in supplementary material 1.

Using the taxonomy given to these OTUs, we manually curated a trait database of 13 morphological and trophic features gathered from the literature, including SizeMin, SizeMax, Cell-Cover, Cell-Shape, Presence of Spicule, Cell-Symmetry, Cell-Polarity, the ability to form Colony, Motility, Ingestion method, Symbiosis type, and the ability to form a Resting-Stage during the life cycle [24, 27]. The taxonomy attributed to these OTUs represented 1680 taxonomic references, due to many OTUs being annotated either with the same species name or with the same unresolved taxonomy. Traits could be annotated to 1380 distinct taxonomic references, representing 41 614 OTUs and 1.06 × 10^7^ reads (82% of the taxonomic references, 46% of the OTUs and 47% of the reads from the whole dataset). For most protistan Divisions, trait annotation was performed for > 50% of the total number of OTUs (except for Division *Rhizaria*, with only 35% of OTUs annotated), see supplementary Table 1 for more details.

### Defining rarity

To investigate rarity patterns at the meta-community scale, we computed rank-abundance-curves using OTUs’ total read count across all the samples of each size-fraction. Following ref [5], we first defined rare and abundant protistan taxa using the RACs and multivariate cutoffs [10]. More details about this approach can be found in supplementary material 2 and details about the taxonomy of the abundant OTUs of each size-fraction can be found in supplementary Table 2.

We further explored gradients of rarity by dividing RACs into subsets of 5000 OTUs. The subsets start from the most abundant OTUs and go to the rarest, the subsets contain 5000 OTUs and all consecutive subsets contain the last 2500 OTUs of the previous subset. The number of OTUs per size-fraction is not an exact multiple of 5000, the last subsets thus contain less OTUs (e.g. the nano-plankton contains 71 380 OTUs, the last two subsets are *70 000* and *71 380*, they respectively contain OTUs within the following ranks *67 500* to *71 380* (3880 OTUs) and *68 880* to *71 380* (2500 OTUs). This subsetting was performed along the RACs of each planktonic size-fraction.

### Inferring the assembly processes and environmental drivers shaping marine protistan communities

The assembly processes shaping marine protistan communities were inferred following an approach based on phylogenetic and taxonomic turnover [14, 38]. This method assumes phylogenetic signal, i.e. that closely related organisms grow in similar environmental conditions [14]. We thus first tested this hypothesis for the protistan OTUs of our amplicon dataset using the R package ‘iCAMP’ and the set of physical and chemical conditions we detailed previously [38] (see supplementary material 3 for further details). For phylogenetic reconstruction, OTUs sequences (V4 region of 18 S rDNA) were first aligned with MAFFT [v7.407] [39], and the phylogenetic tree was constructed with FastTree [v2.1.10 SSE3], following the pipeline of ref [40]. Assembly processes were inferred following the procedure of references [14, 15]. The pipeline first computes: (1) the ß-Nearest-Taxon-Index (ßNTI), calculated as the difference between the observed and the mean of 999 null models of ßMNTD (a metric of phylogenetic turnover computed across pairs of samples). Null models were computed using random shuffling of the branches of the phylogenetic tree). (2) The Raup-Crick metric (RC) is computed as the difference between the observed and the mean of null community turnovers using Bray-Curtis distance. The assembly process explaining the difference between each pair of community is then inferred based on ßNTI and RC, following the algorithm of ref [15].: |ßNTI| > 2 is interpreted as a dominance of *selection*, |ßNTI| < 2 and |RC| > 0.95 is interpreted as a dominance of *dispersal* and |ßNTI| < 2 and |RC| < 0.95 is interpreted as a dominance of *ecological drift* or *stochasticity*. Negative values of ßNTI and RC represent communities that are more similar than expected by chance; selection, and dispersal are thus considered ‘homogenous’ or ‘homogenizing’, at the contrary positive values represent communities where ‘variable selection’ and ‘dispersal limitation’ favor dissimilarity or heterogeneity (higher than expected by chance).

Assembly processes were inferred for the subset of abundant OTUs, and for the subsets of RACs. Because rare OTUs tend to occur in fewer samples (supplementary material 1), the number of samples used to infer the assembly process is reduced towards the rare end of RACs. We note this could affect the relative importance of the different assembly processes, e.g. by affecting the geographic range of the set of samples and thus dispersal limitation. However, the reduction was not drastic (from 257, 325, and 290 to 249, 308, and 272 respectively for micro, nano, and pico-plankton), we thus included these subsets in the analysis. Assembly processes were also inferred across protistan Divisions (broad taxonomic level), by using only the subset of OTUs part of each division and based on their distribution along the samples of each size-fraction (divisions containing less than 20 OTUs in a size-fraction were discarded).

We analyzed whether some of the environmental factors measured in our survey could explain the assembly processes (supplementary material 4). We performed a Permutational Multivariate Analysis of Variance between the phylogenetic turnover (ßMNTD) and our set of environmental parameters (PERMANOVA; ‘adonis()’ function of R package ‘vegan’ [41]).

### Correlation between rarity, traits, and phylogeny

Using traits and phylogeny, we studied whether the rare protistan biosphere displayed specific phylogenetic or functional patterns. We first tested whether OTUs closely related in terms of phylogeny were also functionally similar. In order to do this, we studied the correlation between the phylogenetic and functional distance among pairs of OTUs. The computation of complete distance matrices was hindered by the large number of OTUs involved, e.g., 41 614 OTUs annotated with functional traits would amount to a distance matrix of 1.73 billion cells. To overcome this, we created 999 random subsets of 100 OTUs from which we computed and compared the phylogenetic and functional distance matrices. The phylogenetic distance matrix was computed in R following ref [42]., using OTUs V4 sequences and the R packages ‘DECIPHER’ [43] and ‘phangorn’ [44] (function ‘dist.ml()’ with default parametrization). The functional distance matrix was computed using our database of 13 morpho-trophic traits and Gower’s distance [45]. The correlation between the two distance-matrices was computed using the correlation coefficient of Pearson (R^2^ and associated p.value) and linear regression. We present the average correlation coefficient and linear regression of the 999 random subsets of 100 OTUs.

To study whether the rare biosphere displayed a phylogenetic signal, the correlation between rarity and phylogeny was investigated following the same procedure, using OTUs’ total read abundance and rank per size fraction (both converted in Euclidean distance matrices; see supplementary material 5).

To study functional diversity along RAC, we used the subset of OTUs functionally annotated (41 614 OTUs). In a supplementary work, we first quantified functional diversity along RACs using the R package ‘mFD’ [46] (see supplementary material 6). We also quantified the number of taxa annotated with traits along the RACs of each size fraction (supplementary Table 3), and confirmed that the quality of annotation remained homogeneous along RACs. Then, to delineate potential ecological strategies between abundant and rare protists we studied the correlation of OTUs’ total abundance, rank, and occurrence per size-fraction with each of our 13 traits. Each categorical trait is sorted into ordered categories; e.g. the modalities of the trait *Motility* were sorted into the following order: *Attached* < *Floating* < *Gliding* < *Swimming*, supposing this order represents a growing investment in motility (the order of modalities for all different traits is detailed in [24]). Because most traits are categorical, we used Spearman’s correlation for this analysis. To get the specific signal of each size fraction, OTUs were sorted into the size fraction in which they were the most abundant and excluded from the others. The results from this analysis were congruent with results from the same analysis without the OTU-sorting step.

### Effect of the phylogenetic resolution

Aware that the phylogenetic resolution of our study could affect our results [47, 48], we generated a second dataset in which the Swarm2-OTUs were further clustered at 95% of identity using ‘vsearch’ (--id 0.95) [49]. We reproduced: (1) the RACs per-size fraction and subsets of 5000 OTUs (see section *Defining rarity*), (2) the computation of assembly processes and PERMANOVA (see section *Inferring the assembly processes…*), and, (3) the analysis of the link between phylogeny and abundance (see section *Correlation between rarity, traits, and phylogeny*). The results of these analyses can be found in supplementary material 7.

## Results

### Amplicon sequencing data and defining the rare biosphere

Our amplicon sequencing survey failed to saturate protistan diversity (96% of the estimated diversity, supplementary material 1), but our approach performed better than other large-scale studies; e.g., a survey of protists from European coastal ecosystems (64% of the diversity covered) [12], the open-ocean (75%) [35], or neo-tropical rainforests (unsaturated accumulation curves) [50]. We also acknowledge that other pipelines based on denoising might have performed better in terms of diversity saturation (e.g., DADA2), but at the cost of detecting less rare taxa, see ref [51]. Out of the 90 432 OTUs retrieved, 44 813, 71 380, and 51 568 OTUs were found in micro, nano, and pico-plankton, respectively; 58 395 OTUs were shared across size fractions, while 9 158, 15 446, and 7 433 were specific to micro, nano and pico-plankton. The nano-plankton was the richest fraction (> 70 000 OTUs), probably due to the larger sampling effort in this size fraction (325 compared to < 300 samples in other fractions).

The RAC of each size-fraction displayed a typical power-law decay [12, 52] (supplementary material 2), which indicated that most of the protistan diversity was rare. Using the multivariate approach of refs [5, 10], we detected 38, 32, and 40 abundant OTUs (supplementary material 2), respectively accounting for 40% (i.e., 2.46 of 6.15 × 10^6^ reads), 55% (i.e., 5.02 of 9.13 × 10^6^ reads) and 55% (i.e., 4.01 of 7.3 × 10^6^ reads) of the total number of reads per size-fraction (micro, nano, and picoplankton). In contrast, there were 44 775, 71 348 and 51 528 rare OTUs in the micro, nano, and picoplankton (supplementary material 2). The larger sampling effort in the nano-plankton was also translated into a higher diversity of rare protists in this size fraction.

### Taxonomic patterns in the rare biosphere

**Fig. 2:**
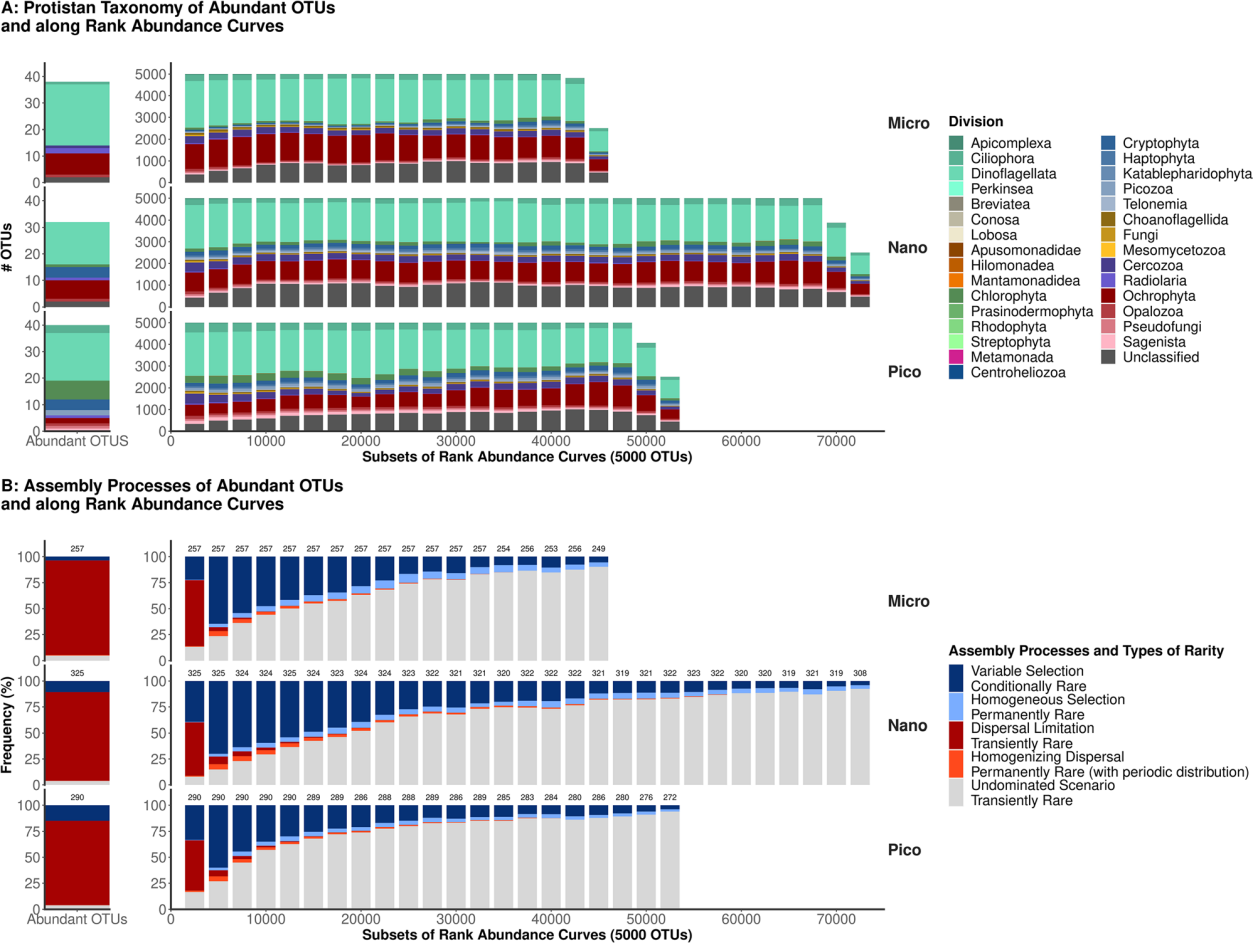
Protistan taxonomic composition and underlying assembly processes along rank abundance curves. Abundant OTUs (left side of all graphs) were selected using the multivariate approach of ref [5]. Subsets of 5000 OTUs were computed with a sliding window along rank abundance curves (X axis on all plots). The last two subsets contain less OTUs because rank abundance curves are not exact multiples of 2500. (A) Taxonomy is given at the Division level (as annotated with the PR2 database v4.13.0 [36]), taxonomic ranks were sorted by Supergroups. (B) Assembly processes were inferred from the distribution of the OTUs in each subset using the method of Stegen et al. [14], the number of samples from which assembly processes were inferred was annotated on top of each barplot. Assembly processes were converted into types of rarity following the nomenclature of ref [5]

To explore rarity as a gradient, we created subsets of RACs. We studied protistan taxonomy among the abundant OTUs and in these subsets. The taxonomy of the 38, 32, and 40 abundant OTUs, their abundance and rank in each size-fractions are summarized in supplementary Table 2. Briefly, OTUs assigned to *Gyrodinium spirale*, *Tripos fusus*, and *Karenia brevis* were the 3 most abundant in micro-plankton (all dinoflagellates). In the nano-plankton, the three most abundant OTUs were *Karenia brevis*, *Gyrodinium spirale* (the same OTUs as in the micro-plankton), and *Heterocapsa sp.* (also dinoflagellate). Whereas OTUs classified as *Ostreococcus lucimarinus*, *Micromonas commode*, and *Bathycoccus prasinos* dominated in the pico-plankton (all part of the Class *Mamiellophyceae*). Among the abundant OTUs, diversity at the division level increased in the smaller size fraction (Fig. 2A).

At the division level, OTU diversity was relatively homogeneous along the RAC and size fractions (Fig. 2A). The division *Dinoflagellata*, notably containing dinoflagellates (class *Dynophyceae*) present in the micro and nano-plankton, and parasitic clade *Syndiniales* primarily present in the nano and picoplankton, dominated the overall diversity with 36.51% of all OTUs in our dataset. *Ochrophyta*, containing diatoms (class *Bacillariophyta*) and other photosynthetic clades, like *Dictyochophyceae* or *Chrysophyceae*, represented 18.20% of all OTUs in our datasets. Also corresponding to a high number of OTUs were the divisions *Ciliophora* (i.e., ciliates; with 5.24% of all OTUs), *Cercozoa* (i.e., flagellates with filose pseudopods; 5.22%), *Chlorophyta* (i.e., usually minute photosynthetic taxa; 3.33%), and *Cryptophyta* (i.e., phototrophic or mixotrophic biflagellates; 2.85%).

The unique taxonomic patterns across size fractions were (Fig. 2A): (1) the higher abundance of pico-sized divisions *Chlorophyta* and *Sagenista* in the smaller size fractions, and (2) the increasing number of unclassified OTUs towards the rarest subsets and across all size fractions (from 7 to 8% to 20–23%).

### Assembly processes and environmental drivers

We then investigated the assembly processes underlying community patterns along RACs. The first major contrast was between the subsets containing the abundant OTUs vs. other subsets; *dispersal limitation* largely dominated the abundant OTUs, explaining 91%, 85%, and 81% of the beta-diversity patterns, while in the subset containing the first 5000 OTUs this process explained 63%, 51%, and 49% of the patterns, respectively for micro, nano, and pico-plankton (Fig. 2B). The influence of this *dispersal limitation* markedly decreased in other subsets, reaching 4%, 7%, and 6% in the second subset (2500–7500) but ranging around 0–1% in rarer subsets (Fig. 2B). Beyond the first 5000 OTUs, *variable selection* was the most dominant assembly process reaching maxima of 65%, 70%, and 60% in the second subset of RAC (2500–7500), in the micro, nano and pico-plankton, respectively. However, the influence of *variable selection* decreased toward the rarest subsets (Fig. 2B), reaching minima of 4–5% in the rare end of the RAC of all size fractions. Turnovers where neither selection nor dispersal were significantly different from the null models, i.e. ‘undominated scenarios’ (Fig. 2B), increased from 13%, 8%, and 16% in the first subsets to 90%, 92%, and 91% in the last subsets of micro, nano and pico-plankton, suggesting more stochastic distributions in rare subsets. Other assembly processes had lower influences, *homogenizing dispersal* peaked weakly in the second subset (2500–7500; 5–6% across size fractions) and reached 0% around the end of RACs. At the same time, while *homogeneous selection* reached in average 5%, 4.6%, and 3.2% within micro, nano, and picoplankton, except in the first subsets where no influence was found. Overall, the three size fractions presented similar patterns in assembly processes along their RAC.

**Fig. 3:**
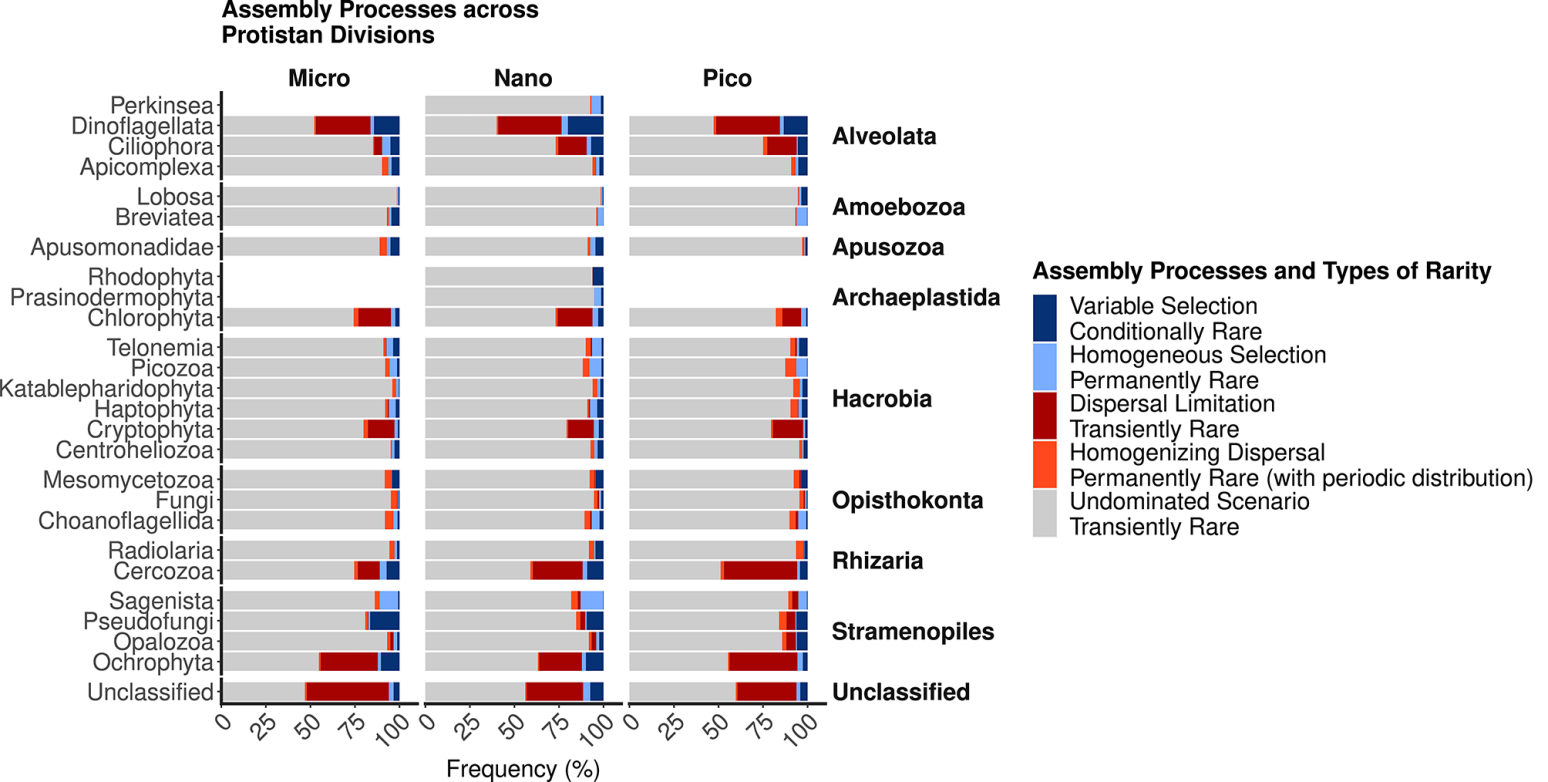
Assembly processes across protistan taxonomic divisions and size fractions. Taxonomic divisions (axis y) were sorted by supergroups. Assembly processes were inferred from the distribution of the OTUs in each taxonomic subset using the method of Stegen et al. [14]. Assembly processes were converted into types of rarity following the nomenclature of ref [5]. Patterns from unclassified OTUs (at the division level) were also computed in each size fraction, although they do not represent a coherent taxonomic group

Studying assembly processes across taxonomic divisions showed that the phyla containing the most abundant OTUs were the most influenced by dispersal limitations (Fig. 3); see e.g. *Dinoflagellata* (30, 36, and 36% for this assembly process in the micro, nano, and pico-plankton), *Ochrophyta* (32, 23, and 38%), *Chlorophyta* (18, 20 and 10%), or *Cercozoa* (12, 28, and 41%). These same divisions also contained OTUs influenced by variable selection, with *Dinoflagellata* (14, 13, and 20% for this assembly process in the micro, nano and pico-plankton) and *Pseudofungi* (16, 9, and 6%) as the divisions the most influenced by this process. Homogenizing selection and dispersal had higher influence on *Sagenista* (these processes explained 13%, 16.5%, and 7% of the patterns from these OTUs respectively in the micro, nano and pico-plankton), *Picozoa* (6.7%, 10.5%, and 12% in micro, nano, and pico-plankton), *Telonemia* (8% in the nano-plankton) and *Choanoflagellida* (7%, 7.5% and 8% in the micro, nano and pico-plankton) than on the rest of the Divisions and community (Figs. 2B and 3). In all divisions, most community patterns were undominated (by either selection or dispersal), illustrating the high prevalence of OTUs with stochastic distributions.

### Rarity, traits and phylogeny of marine protists

**Fig. 4:**
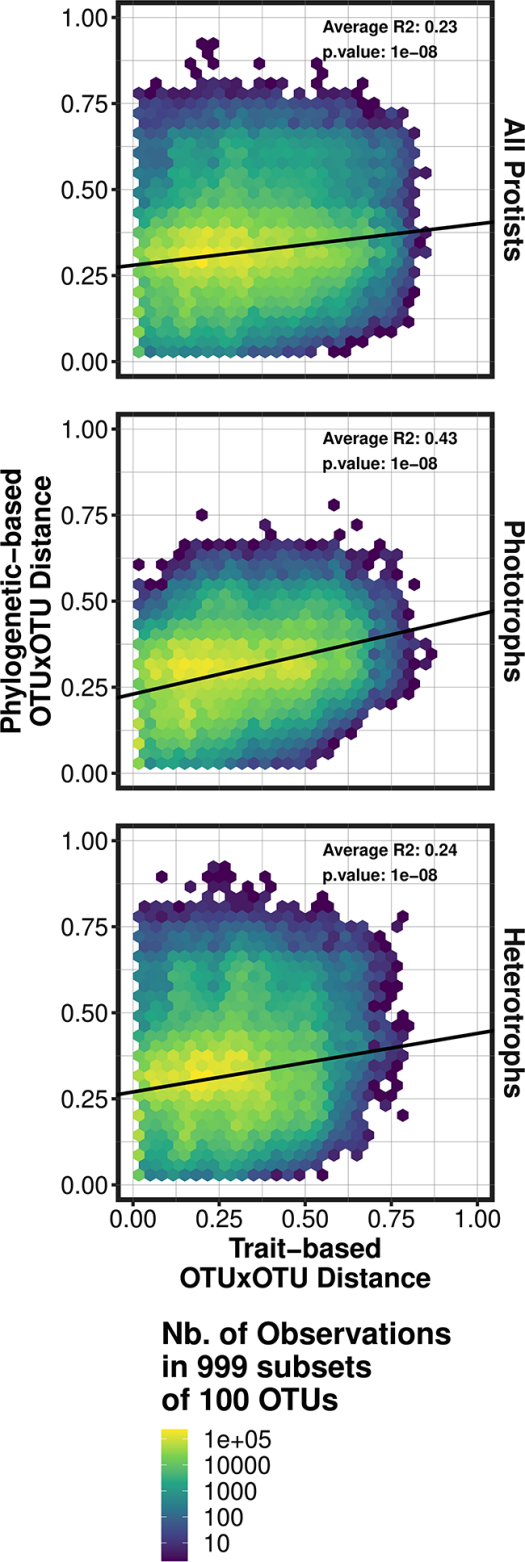
Relationship between protistan phylogeny and traits. Phylogenetic distance between OTUs was computed using the V4 region of the 18 S rDNA and following ref [42]. Trait distance was computed based on OTUs’ traits (annotated with our trait database) and Gower’s distance. The results originate from 999 subsets of 100 OTUs (each subset representing two distance matrices of 10 000 cells). Pearson correlation and a linear model were computed between the two distance matrices of each subset, the coefficients and linear model displayed correspond to the mean coefficients across the 999 subsets. This procedure was repeated for all protists annotated with traits (41 614 OTUs), only phototrophic protists (18 997) and only heterotrophic protists (25 895)

Coherent with the good fit between phylogeny and environmental niches (supplementary material 3), phylogeny and traits were significantly correlated (Pearson’s coefficient of correlation, p.value = 1e-8), suggesting that closely related OTUs feature similar functional strategies (Fig. 4). The correlation between phylogeny and traits was higher for phototrophic OTUs (average R^2^ of 0.43 across subsets) than for all protists (average R^2^ of 0.23) and for heterotrophic protists (average R^2^ of 0.24).

There was no significant correlation between phylogeny and abundance (supplementary material 5), i.e., rare or abundant protistan species were not more phylogenetically related. This pattern was coherent with the low variability of taxonomy along RAC (Division level; Fig. 2A).

**Fig. 5:**
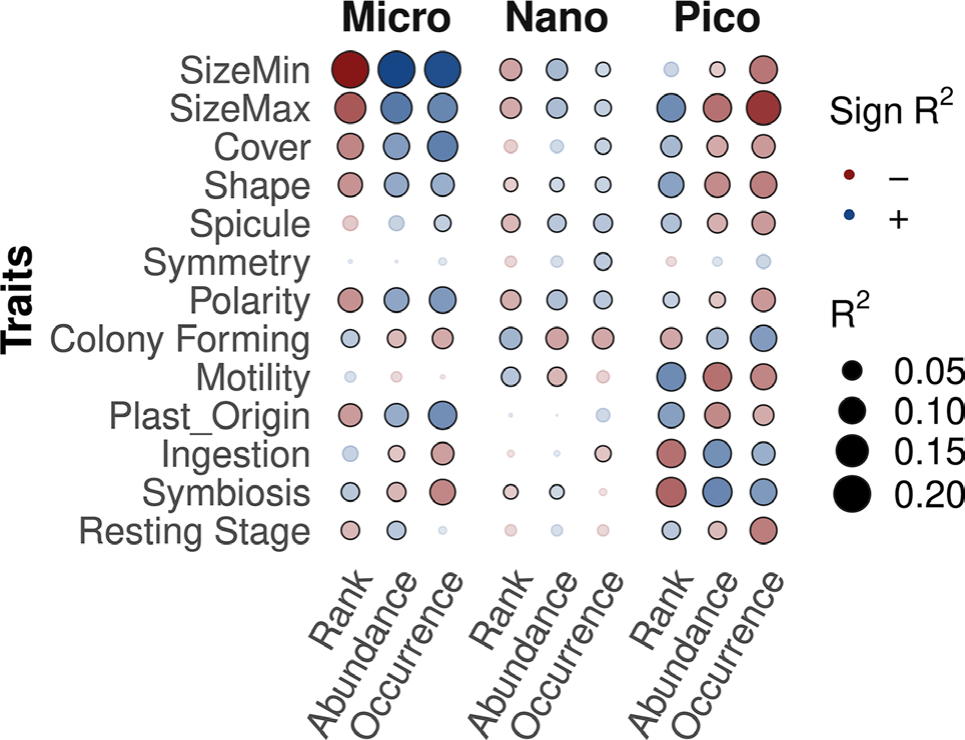
Correlation between OTUs’ traits and their rank in rank abundance curves, total abundance and occurrence across the samples of each size-fraction. Categorical traits were ordered by investment in complexity (details of this ordering can be found in [24]). Correlation was investigated using Spearman’s coefficient, the sign and value of the R^2^ are represented by the color, color intensity, and the size of the bubbles, significant correlations (p.value < 0.05) are circled in black. This analysis could be sensitive to OTUs cross-contamination between size-fractions, OTUs were thus sorted into the size-fraction in which they were the most abundant and excluded from the others

Functional diversity was relatively high throughout the RAC of all size-fractions (supplementary material 6), suggesting that rare protists could be as functionally diverse as abundant ones. We then explored the differences in ecological strategies among rare and abundant protists across size-fractions (Fig. 5). The correlation between the OTUs’ traits, ranks, abundance and occurrence was significant for many traits (p.value < 0.05) but rather low, with absolute R^2^ values ranging between 0.00018 and 0.20 (Fig. 5). The rank, abundance and occurrence of OTUs (X-axis, Fig. 5) generally correlated with the same traits, highlighting that OTUs ranked first were abundant (low rank = high abundance) and occurred in a larger number of samples (supplementary material 1). Correlation with traits differed across size fractions (Fig. 5). The nanoplankton showed strikingly fewer and lower correlations, supposing that, in this size class, traits were distributed randomly along the rarity gradient. However, in the micro and pico-plankton, the highest correlations suggested that abundant and rare protists had contrasting trophic strategies. In the micro-plankton, abundant taxa were generally larger, harbored cell covers, had complex cell morphology (correlation with shape, polarity), dominantly phototrophic (the trait *Plast_Origin* represent a gradient in phototrophy: *None* < *Kleptoplastidic* < *Endosymbiotic* < *Constitutive*) and less prone to symbioses (interpreted from Fig. 5). In turn, this suggested that rare micro-sized protists were small, heterotrophic and harbored less complex cells. In the picoplankton, the roles were reversed, the abundant protists were usually the smallest, with simple morphology, low motility, prone to form colonies, with more heterotrophic, and parasitic lifestyles (*Parasitic* is the highest ranked modality of the trait Symbiosis); while the rare pico-sized protists were consequently larger, motile, with complex morphology, and relying more on phototrophy (Fig. 5).

## Discussion

Using one of the largest collection of marine protistan communities, we aimed to better understand the composition, ecological origin and function of the protistan rare biosphere.

### Do abundant and rare protists differ in composition?

In contrast to a previous survey of marine protists in European coastal waters [12], our survey showed that taxonomy remained stable along the RAC of each size fraction (Fig. 2A). This suggest that the rare biosphere is not composed of specific phyla (at the Division level) and that most protistan Divisions contain abundant and rare species. This result could change at finer taxonomic resolution (e.g. Family, Genus), where lineages include fewer OTUs and have higher chances to occur only as rare or abundant. The increasing proportion of taxonomically unclassified protists among the rare end of RACs (Fig. 2A), could also represent undescribed protists specific to rarity. However, the lack of correlation between phylogenetic distance and abundance (or rank; supplementary material 5), suggests that rare and abundant protists do not constitute evolutionary distant pools of eukaryotic diversity. The discrepancy between our results and those of the previous survey could be explained by under-sampling (only 23 samples vs. 1145 in our study) and low phylogenetic resolution (OTUs clustering at 95% vs. swarm-v2 OTUs, comparable to amplicon variants) [34, 47]. These two factors are likely to result in: (1) the lack of detection of rarer lineages that belong to larger taxonomic groups, and (2) a shallow coverage of microdiversity. By using more samples, our survey presents a higher taxonomic coverage and warrants a higher phylogenetic resolution to further detect microdiversity. This microdiversity is likely to comprise technical biases such as (1) the variability in the detection of microdiversity across taxonomic branches (OTUs at genus, species or population level depending on the taxa), (2) variants generated during sequencing, and (3) the difference in the number of marker DNA copies per cell, which could bias abundance estimation (however, the number of copies per cell should be more homogeneous among size-fractions) [48]. The lack of correlation between phylogeny and abundance persisted when clustering our OTUs at 95% of identity (supplementary material 7). We thus suggest that microdiversity (defined at multiple levels) homogenizes taxonomic composition along RACs. Ecologically speaking, this microdiversity is likely to play a key role in maintaining community functions [53] or strain-specific interactions [54] under varying environmental conditions. This pattern is likely to be common to all microbial communities.

### Which assembly processes and types of rarity lead to the formation of the protistan biosphere?

Studying the assembly processes driving the rare microbial biosphere can reveal the different strategies of rarity adopted by microbes [5, 14, 55]. To quantify the assembly processes shaping marine protistan communities, we used a method that assumes that phylogeny is a good predictor of niche (the set of conditions where an organism can grow) divergence [15]. We first successfully verified the existence of a phylogenetic niche conservatism among marine protists, suggesting that closely related protistan taxa have similar environmental niches [29, 56].

Across size fraction, the distribution of abundant protists was only limited by dispersal, while the distribution of rarer taxa was driven by selection and stochasticity for the rarest taxa (Fig. 2B). These results bear similarities with other surveys on freshwater bacterioplankton [57], marine pico-eukaryotes [58] or soil fungus [59]. It has been suggested this pattern stems from habitat specialization, where abundant microbes tend to be more generalists; they can grow in most of the ecosystems they can be dispersed to; while rarer OTUs are habitat specialists; they can only grow in a limited set of conditions and thus respond more to selection, and have narrower distributions [60]. Coherent with this theory, abundant OTUs tended to occur in a more samples in our survey (supplementary material 1). Stochasticity, as observed in the rarest subsets of RAC, has been reported to be more preeminent among rare protists [61, 62]. This is likely due to most approaches, ours included, being unable to infer assembly processes from OTUs with very limited coverage (low abundance and occurrence across samples). Our results also probably reflect the scale of our survey, with high habitat connectivity for example, studies at the global scale showed a higher contribution of dispersal limitation for oceanic pico-eukaryotes [63], and more stochastic patterns in rare benthic micro-eukaryotes [61]. In these large-scale surveys, communities are less connected, and rare taxa are more likely to occur only once, contributing to a higher importance of stochastic processes compared to selection [15, 47]. The prevalence of selection is also likely to vary according to the type, the variability and the intensity of selective pressure within the ecosystem in which it is studied [58, 64, 65], e.g., protistan communities in O_2_-depleted bottom marine waters showed a stronger influence of selection than in surface waters [64].

Rare taxa prone to *variable selection* have been described as *conditionally rare taxa*, maintained at low abundance but able to become prevalent when favorable changes in selective pressure occur through time or space [5]. They dominated intermediate levels of rarity in our survey (*Variable Selection* in Fig. 2B). These conditionally rare protists may represent the many subpopulations of abundant taxa that compose microdiversity [53, 54]. Due to low sinking rates [66], pico-eukaryotes can easily persists in the water column as conditionally rare organisms. Some other larger protists might rely on their ability to form dormant stages that will sink on the seafloor [67, 68], and wait to be re-suspended in suitable conditions. This mechanism has been termed the “microbial conveyor belt”, where taxa transition, through time and space, between the rare and abundant sub-communities using dormancy in unsuitable environments and growth when resuspended in better-suited conditions [69]. However, this “conveyor belt” and the high importance of *conditionally rare taxa* may be specific to shallow aquatic systems, as resuspension in deeper ecosystems is less frequent. In accordance, protists from the open-ocean generally form less resting stages [68]. In turn, rare taxa that occur stochastically and in low numbers have been called *transiently rare taxa*. They have been interpreted as products of failed migration or diversification, detected occasionally but undergoing extinction in the ecosystem surveyed [5]. Their high proportion in the rare end of RACs (see *Undominated Scenario*, a proxy of stochastic distribution, in Fig. 2B), may be due to the gathering of transiently rare protists from several communities within a meta-community (Fig. 2B). Transiently rare protists in coastal ecosystems could originate in migrations from the neighboring terrestrial, freshwater, benthic, or oceanic systems with low success rate, followed by extinction in the new environment (i.e. mass-effects) [70, 71]. Most interestingly, the small part of the rare protistan biosphere influenced by *homogenizing selection and dispersal* could represent *permanently rare taxa* (Fig. 2B). This could happen to communities containing rare taxa using resources that are constantly available over time and space, or to taxa brought with a stable flux from another ecosystem [20]. These taxa might harbor adaptations to rarity, thus avoiding predation or viral infections at the cost of a narrower niche and lower growth rate [5, 20, 55]. Some *permanently rare taxa* have also been hypothesized to be “keystone”, being involved in multiple interactions and specific functions performed within microbiomes [3, 62]. We evidenced that some OTUs within *Sagenista*, *Picozoa*, *Telonemia*, or *Choanoflagellida* could follow this strategy (Fig. 3). These OTUs could represent sub-groups among protistan Divisions that are exclusively rare (as suggested by our hypothesis that at the finer phylogenetic resolution, we could identify exclusively rare lineages). These divisions contain poorly studied bacterivores and decomposers with intricate feeding strategies [72]. By being rare but occurring in many samples, these taxa are likely to play an under-appreciated role in the microbial loop that allows the recycling of organic matter (either specific compounds or specific bacterial preys) in aquatic ecosystems [22]. The existence of such taxa also underlines that specific functions might only be performed within the rare protistan biosphere [2, 3].

The contribution of assembly processes changes according to the ecosystem surveyed, the scale at which it is studied, and the microbial compartment under investigation [20]. However, our results were conserved when clustering our OTUs at 95%, suggesting that the phylogenetic resolution does not affect these patterns (supplementary material 7). We thus hypothesize that given a distance and an environmental variability sufficient for *dispersal limitation* and *variable selection* to occur, other microbial communities might be composed of the same ecological strategies along RACs: (1) abundant, generalists, taxa whose distribution is only regulated by dispersal, (2) *conditionally rare taxa* selected during favorable conditions in intermediate levels of rarity, and (3) *transiently rare taxa* migrating stochastically from neighboring ecosystems but going extinct locally (mass-effect), composing the rare-end of RACs. The contribution of *permanently rare taxa* is likely to change with the scale and grain of the survey, e.g. if the same community is sampled repeatedly, a rare taxon will be categorized as permanently rare, but if this community is sampled only once within a larger context, the same rare taxon will be considered *transient* (if not occurring elsewhere) [20, 47]. A good perspective to decipher permanently and transiently rare taxa could be to study their activity via RNA-based approaches, with taxa under extinction (i.e. transiently rare) likely showing little to no activity [19]. Further investigations are required to decipher the link between ecological scale (spatial, temporal and phylogenetic), assembly processes and rarity types among microbial communities.

### Exploring the functional diversity of the rare protistan biosphere

After investigating the taxonomic and phylogenetic composition of the rare protistan biosphere, we studied its functional diversity using of a trait approach. The correlation between traits and phylogeny was significant across size fractions (Fig. 3), suggesting that morphological traits have been conserved across evolution, such that closely related protistan phyla show similar traits sets. Phylogeny was already known to fit well with metabolic traits among phototrophic protists traits [73], and the congruence between phylogeny, metabolic traits and morphology points to laws of allometry [66]. The fit was weaker for heterotrophs, (Fig. 3), suggesting that the chance of distantly related phyla harboring the same traits was higher in this group. We hypothesize that this is not an emergent pattern but the consequence of the difficulty in correlating the grazing activity of heterotrophs (rate and specificities) with morphological or even genomic traits [74]. It thus remains to be investigated whether the functional diversity of heterotrophs follows phylogeny.

The poor fit between traits and the abundance of OTUs (Fig. 5) suggested that traits were widely distributed along RAC. This supports the hypothesis of high functional redundancy within the rare protistan biosphere. This also aligns with the rare biosphere comprising high microdiversity, with rarer subpopulations harboring the same functional potential as their abundant counterparts. We note that our approach, comprised of traits measurable across all protists, might exclude traits that could be specific and unique to the rare biosphere [3]. Approaches relying on taxonomic annotation might also perform less well than approaches based on molecular homology, however these methods have yet to consider the full spectrum of the eukaryotic functional diversity [75]. Redundancy in this broadly categorized functional diversity is nevertheless relevant for the ecosystem. Functionally rare redundant protists are likely to replace their abundant counterpart during disturbances or changes in the ecosystem, thus allowing the maintaining of ecosystem functioning [8, 53].

In addition, we identified trends in the dominant trophic strategy in micro and pico-plankton (Fig. 5), with abundant phototrophs and rare heterotrophs (i.e. phagotrophs and parasites) in the micro-plankton. At the same time, heterotrophy was generally abundant, and phototrophs correlated negatively with abundance in the pico-sized plankton (Fig. 5). Before discussing this result, some technical aspects should be considered. Mixotrophy, supposed to be widespread among marine protists [76], is largely underestimated by our trait approach due to a lack of referencing in the literature [24]. In the pico-plankton, phototrophs dominate coastal pico-eukaryotic communities in terms of cell numbers [77] (as also evidenced in supplementary Table 2). The correlation between phototrophy and abundance is probably weakened by the fact that phototrophs have representatives thoughout the rank abundance curve of pico-plankton. In turn, pico-heterotrophs may have fewer representatives that are mostly abundant.

Nevertheless, we hypothesize that the distribution of trophic strategies along these size fractions could be an emergent property of protistan food webs. In the eutrophic coastal systems surveyed, large phototrophic protists with higher maximal nutrient uptake abilities can bloom in profusion [78, 79], which relegates large heterotrophs to rarity [32]. This in turn affects the smallest size-fraction, where small heterotrophs are rather successful and abundant by benefiting from the production of larger organisms [77]; either by parasitizing them, feeding on the detrital organic matter they produce or on the bacteria growing to recycle this organic matter [22]. In more oligotrophic systems like the open-ocean, primary production is primarily carried out by pico-sized cells which are more efficient at acquiring nutrients in oligotrophic systems due to a higher surface to volume ratio and smaller diffusion boundary layer [66, 79]. In these conditions, large phototrophs would be rarer, whilst large heterotrophs predating on pico-phototrophs could reach high abundances [80]. We thus hypothesize that the distribution of trophic modes along the size spectrum is not fixed [79, 81], but is likely to be dynamic and to shift according to the most available resource for marine protists (i.e. light, nutrients or organic carbon) [82, 83]. The magnitude of this process could be investigated with methods measuring the CO2 balance of the different size fractions across a gradient of trophic regime [80, 84], but this is beyond the scope of our study.

## Conclusions

In summary, on a regional scale, rare marine protists within connected coastal ecosystems were not taxonomically, phylogenetically, or functionally different from their abundant counterparts. This is coherent with the hypothesis of a high functional redundancy within the rare protistan biosphere, which could help protists buffer future environmental changes. Studying the assembly processes acting upon these protistan communities, we found that the environment affected less abundant protists, whose distribution was driven mainly by dispersal limitations. In turn, rarer protists were (1) more influenced by selection, highlighting the existence of *conditionally rare taxa*, or (2) had stochastic distributions, which reflected the influence of mass effects from neighboring ecosystems. Some taxa with poorly characterized functions were rare across all communities, suggesting that the rare protistan biosphere could also play an underappreciated role in aquatic systems. Finally, the balance between phototrophy and heterotrophy changed along the RAC of different size fractions, suggesting an interplay between protists of various sizes in eutrophic conditions. Further exploration of the rare microbial biosphere is required to better predict how microbial communities shape ecosystems, be they natural, future or engineered.

### Electronic supplementary material

Below is the link to the electronic supplementary material.

Supplementary Material 1

Supplementary Material 2

## Data Availability

The amplicon sequencing data generated and analyzed in the current study (raw, filtered, and clustered) is available in the following repository: https://data-dataref.ifremer.fr/bioinfo/ifremer/dyneco/POHEM/. The work on the trait annotation of the OTUs of this dataset is available in the Seanoe repository: https://www.seanoe.org/data/00405/51662/.
