# Supplementary material for: Assembly processes and functional diversity of marine protists and their rare biosphere
Source: Environ Microbiome. 2023 Jul 13;18:59. doi: 10.1186/s40793-023-00513-w (PMC10347826; doi:10.1186/s40793-023-00513-w)
Supplement: Supplementary file 2 — Supplementary Material 2 [file 40793_2023_513_MOESM2_ESM.pdf]

Supplementary Material and Figures

Title: Assembly processes and functional diversity of marine protists and their rare biosphere

Authors and affiliations: Pierre Ramond[1*], Raffaele Siano[2], Marc Sourisseau[2], Ramiro Logares[1]

[1]Institute of Marine Sciences (ICM), Department of Marine Biology and Oceanography, CSIC, 08003 Barcelona, Catalunya, Spain

[2] Ifremer-Centre de Brest, DYNECO/Pelagos, Technopôle Brest Iroise, 29280 Plouzané, France

*** Corresponding author**: email: pierre@icm.csic.es; address: Institute of Marine Sciences (ICM), CSIC, 08003 Barcelona, Catalunya, Spain.

Competing Interests: The authors declare no competing interests.

Supplementary Material 1: Complementary diversity analysis.

OTUs' total read abundance was calculated across our complete dataset, for the three size-fractions (micro, nano and pico-plankton) and across the years covered by our survey (2009-2015). The rarefaction curves as well as diversity extrapolations were then computed with function *iNEXT(datatype="abundance")* from R package iNEXT [1]. The results are summarized in the Figure S1A below. We also investigated the relation between the abundance and occurrence of all OTUs across the samples of each size-fraction (Figure S1B). Finally, we studied OTUs distribution across size-fractions with a Venn diagram (Figure S1C).

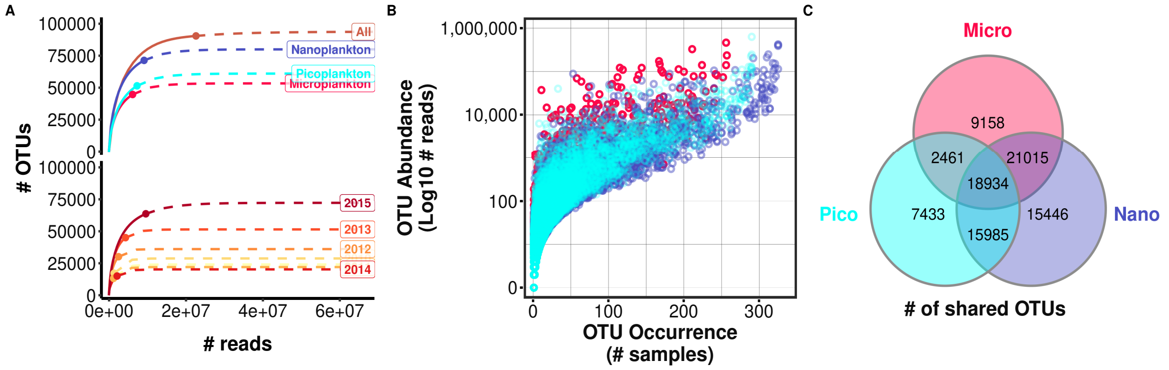

Figure S1: Complementary diversity analyses. A) Rarefaction curves for our complete dataset and across size-fractions (top plot), as well as across years (bottom plot). The solid lines correspond to the interpolated rarefaction curves computed by iNEXT, the circles correspond to the observed number of reads and OTUs, the dashed lines represent the extrapolated rarefaction curves computed by iNEXT. B) Relation between OTUs' abundance and occurrence across samples, each circle corresponds to an OTU. Abundance, estimated by the total number of reads of an OTUs across the samples of each size-fraction, was log10 transformed for better representation. C) Venn diagram of the presence of OTUs across three size-fractions.

The asymptote of a rarefaction curve is used as a proxy for diversity saturation. Across our complete dataset, the three size-fractions and years, rarefaction curves did not reach diversity saturation (Figure S1A). Diversity extrapolation indicated that diversity in our dataset would amount to ca. 93 500 OTUs, which based on our approach would require 5.5×10^7 reads. However, our dataset represents a very good share of this diversity, as we retrieved 90 432 OTUs, i.e. 96% of the estimated diversity. It is likely that the portion of diversity that was missed by our approach corresponds to very rare OTUs with very low abundance. Saturation depended on the number of samples included, we thus decided to work on the largest subsets of dataset possible (per size fractions), and decided against further sub-division of our dataset (across years of spatial regions). Although there existed high variability, we observed that abundant OTUs tended to occur in a larger number of samples, while rarer OTUs occurred in less samples (Figure S1B). It is thus likely that the rare portion of diversity that was missed by our approach would occur in very few samples. The Venn diagram (Figure S1C) indicated that most OTUs were found in several size-fractions. As expected adjacent size-fractions (micro-nano: 21 015 shared OTUs, and nano-pico: 15 446 shared OTUs) shared more OTUs than non-adjacent size-fractions (micro-picoplankton: 2461).

Supplementary Material 2: Defining rare and abundant OTUs based on Multivariate Cutoff Level Analysis.

We defined rare and abundant OTUs across size-fraction following the method detailed in references [2,3]. This approach studies the correlation between the original distance matrix of a dataset (all pairwise samples' comparison) and distance matrices computed on subsets of the community from which an increasing number of rare OTUs are removed. In this approach, rare OTUs are those OTUs from which the removal does not affect the correlation between the original and subset distance matrices. The Bray-Curtis distance was used to infer distance matrices from the original and subset datasets [4,5]. The correlation between the original and subset distance matrices was inferred using Spearman's rank correlation and Mantel test, both proxies range between 1 (good correlation) and 0 (poor correlation). Across the rank abundance curves of all size-fractions correlations below 0.9 steeply decreased (Figure S2C), we thus choose this values as the cutoff above which the removed OTUs were considered rare (and the OTUs left as abundant). The subsets correspond to even removals of reads from the total read abundance, i.e. we removed the amount of rare OTUs from which the abundance corresponded to a given percentage of reads.

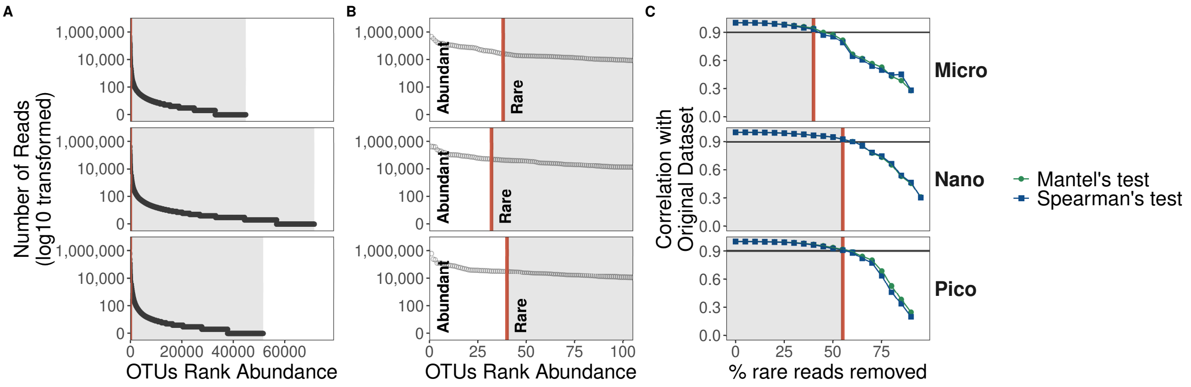

Figure S2: Rank abundance curves of all size-fractions and multivariate definition of rarity. Rank abundance curves In Figure S2A were computed based on the number of reads of each protistan OTUs across all the samples included in each size-fraction, each OTU is represented by a circle. Figure S2B represent a zoom on the first 100 OTUs of all rank abundance curves. The multivariate definition for rarity presented by Figure S2C relies on the correlation (Mantel or Spearman tests) between the Bray-Curtis distance of the original dataset (one for each size-fraction) and the distance within subsets of this dataset from which an increasing number of rare reads and OTUs are removed. When the correlation reaches values under 0.9, the OTUs left are considered abundant while the OTUs removed are considered rare. This threshold value is represented as a red line across plots and the following gray area represents rare reads or OTUs.

The multivariate method (Figure S2C) highlighted that there were 38, 32 and 40 abundant OTUs, respectively accounting for 40% (2.46 of 6.15 × 10^6 reads), 55% (5.02 of 9.13 × 10^6 reads) and 55% (4.01 of 7.3 × 10^6 reads) of the total number of reads per size-fraction (micro, nano and picoplankton; Figure S2A &B). Reversely, there were 44 775, 71 348 and 51 528 rare OTUs in the micro, nano and picoplankton (Figure S2A).

Supplementary Material 3: Testing the phylogenetic signal of niche divergence.

This section was performed using functions from R package 'iCAMP', parametrization of the commands followed the recommendations from the supplementary material of the original iCAMP paper [6]. This procedure was performed per size-fraction, using samples for which environmental conditions were available (Temperature, Salinity, NH4, NOX, PO4 and SiOH4). For this test, we selected only OTUs present in at least 10 different samples per size fractions, considering this threshold as the minimum number of occurrence to give a reliable estimate of environmental niche. The test was performed for 3106, 6410 and 4041 OTUs, across 191, 254 and 238 samples, respectively for micro, nano and pico-plankton.

The procedure of the test is:

1) A distance matrix of niche values is computed for the OTUs of each size-fraction and for each environmental variable with the function 'dniche(method = "niche.value")'.
2) A phylogenetic distance matrix is computed for the OTUs of each size-fraction with the function 'pdist.big()'.
3) Based on each phylogenetic distance matrix, phylogenetic bins (containing phylogenetically similar OTUs) are computed with function 'taxa.binphy.big(ds = 0.2, bin.size.limit = 24)'. This command generated 51, 118 and 70 phylogenetic bins, respectively for the OTUs of micro, nano and pico-plankton.
4) For each phylogenetic bin of each size-fraction, the correlation between the phylogenetic distance and the niche distance matrices are compared with function 'ps.bin(cor.method = "pearson")', using Pearson's Correlation coefficient. The output of this command is a coefficient of correlation (R^2) between each phylogenetic bin and environmental variable, with the associated p.value.

Overall, there existed a significant correlation between protistan phylogeny and environmental variables within most bins in all size-fractions (40 of 51, 91 of 118, and 51 of 70 phylogenetic bins, within micro, nano, and picoplankton, respectively), thus warranting the use of phylogenetic turnovers for investigating the assembly processes driving the community patterns in our dataset.

Supplementary Material 4: Testing the fit between environmental variables and assembly processes.

We studied whether some of the environmental factors measured in our survey could explain the assembly processes of marine protists along subsets of the ranbk abundance curves we built for each size-fraction. we performed a Permutational Multivariate Analysis of Variance between the phylogenetic turnover (ßMNTD) and our set of environmental parameters (PERMANOVA; ‘adonis()’ function of R package ‘vegan’ [7]). The variables were not co-varying (Pearson correlation between variables < 0.8), all environmental factors measured were thus included in this analysis. The PERMANOVA’s R2 is a proxy of how well the environmental variables fit with the ßMNTD.

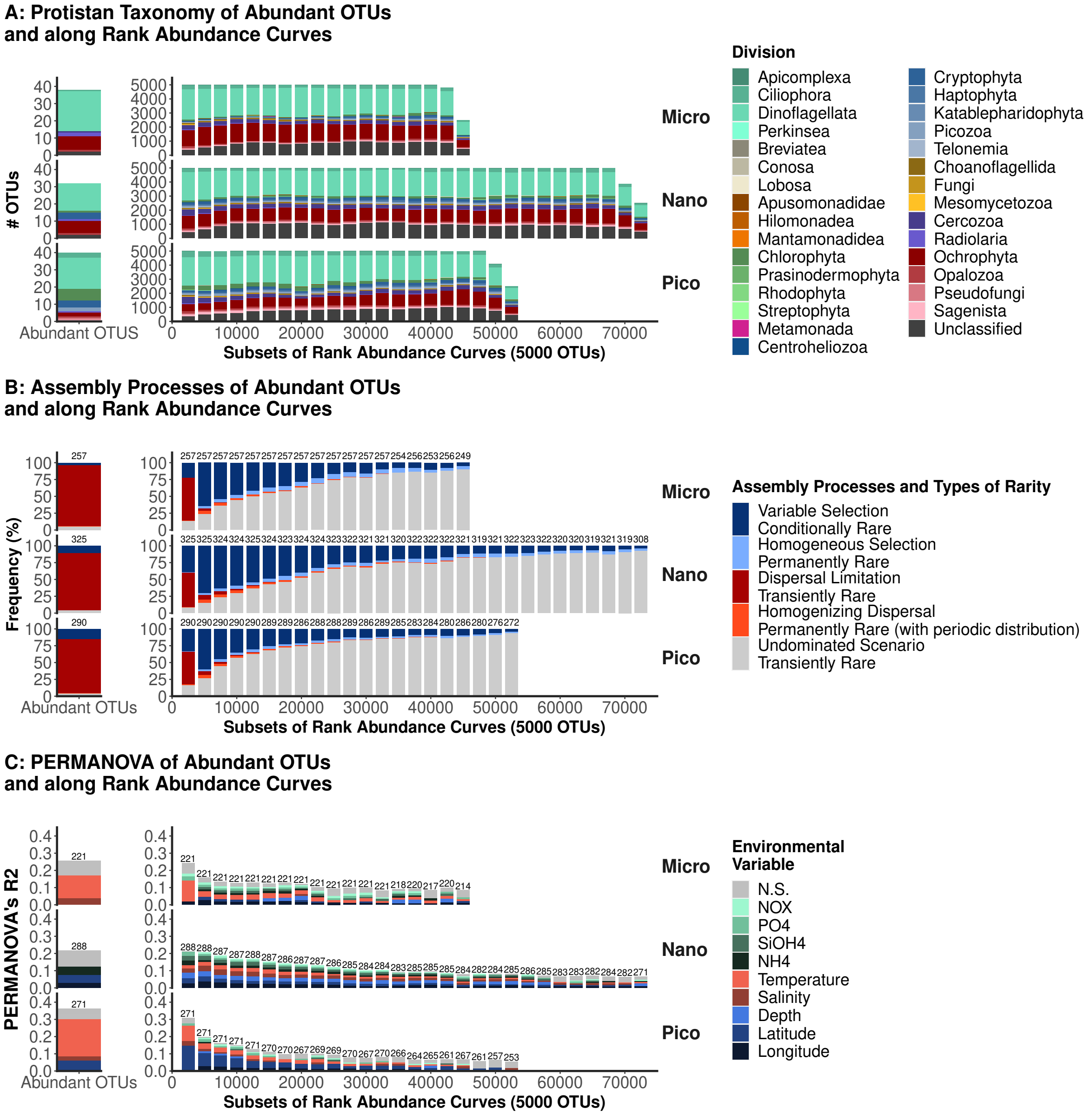

Figure S3: Protistan taxonomic composition, underlying assembly processes and environmental drivers along rank abundance curves. Abundant OTUs (left side of all graphs) were selected using the multivariate approach of ref. [5] . Subsets of 5000 OTUs were computed with a sliding window along rank abundance curves (X axis on all plots). The last two subsets contain less OTUs because rank abundance curves are not exact multiples of 2500. A) Taxonomy is given at the Division level (as annotated with the PR2 database v4.13.0), taxonomic ranks were sorted by Supergroups. B) Assembly processes were inferred from the distribution of the OTUs in each subset using R package iCAMP ref. [6], the number of samples from which assembly processes were inferred was annotated on top of each barplot (the same procedure was applied in C). Assembly processes were converted into types of rarity following the nomenclature of ref. [3]. C) PERMANOVAs were computed using the ßMNTD of each subset (computed during the inference of assembly processes) and the environmental variables measured in our survey. The barplots represent the total R^2 of each PERMANOVA and the color code represent the contribution of each variable to the fit. The overall fit sometimes includes variables for which the individual fit is non-significant (see the gray color code, N.S. stands for not-significant), not to be confused with residual correlation (= 1 – overall fit).

The assembly processes (Figure S3B) fitted significantly with different environmental variables across size-fractions (Figure S3C). The fit ranged between 0.05 and 0.36 across subsets (value of the R^2). The explaining power of environmental variables peaked within the subsets containing the most abundant OTUs and decreased towards the rare-end of rank curves (Figure S3C). It also varied across size-fractions with maxima of 0.25, 0.21, and 0.36 in the micro, nano, and pico-plankton. Micro-plankton appeared to be mostly driven by physical (temperature and salinity) and chemical conditions (nutrient concentrations); Nano-plankton was driven evenly by physico-chemical conditions and distance, both geographic (latitude and longitude) and bathymetric (depth); Pico-plankton was mostly driven by physical conditions (temperature) and geographic distance (Figure S3C). Overall, the set of environmental conditions driving OTUs remained the same along RAC, with few exceptions, e.g. the influence of depth on OTUs in the ranks 15 000 to 20 000 in the micro-plankton (Figure S3C).

The environmental variables measured in our study thus explained only a small fraction of the variation of community patterns across size-fractions, suggesting that un-measured variables (e.g. biotic) or legacy effects (i.e. lags between shifts in community and the environment) could have a large importance on the assembly of marine protists [8]. We hypothesized that the explaining power of environmental variables would peak in subsets where variable selection would dominate the assembly processes and decrease in subsets driven by stochastic processes (Figure S3B). Counterintuitively the explaining power peaked in the most abundant subsets where dispersal limitation dominated (Figure S3B). However, variables involved in dispersal limitation explained most of the fit in these subsets, as

temperature, reflecting thermal boundaries between water masses (fronts or thermoclines) and seasonal contrasts [9], and geographic distance, directly related to dispersal, were the main explaining variables for abundant OTUs (Figures S3B and S3C). The explaining power was thus higher for the communities in these subsets because they were driven both by dispersal and selection. Among intermediate levels of rarity, from the start to the middle of the RAC where variable selection was more pro-eminent (Figure S3B), nutrients had a larger influence for the micro and pico-plankton. Variability in nutrient concentrations is indeed a strong selection factor for phototrophic microbes [10], which constitute a large share of coastal protistan communities [11]. Ammonium (NH_4^+) is a proxy of the remineralization performed by heterotrophic protists within the microbial loop [12,13], but it is also a resource, preferably for small phytoplankton in poor light conditions [14]. The good fit between ammonium concentration, water depth (linked to light availability) and the most abundant protists of the nano-plankton thus stresses the role of this size-class on remineralization and the uptake of ammonium. Finally, pico-sized protists showed a higher fit with geography, which could be explained by a generally narrower geographic distribution compared with larger size-classes [15,16].

Supplementary Material 5: Phylogenetic signal of rarity.

We tested whether OTUs closely related in terms of phylogeny were also close in abundance and within rank abundance curves. To do this, we studied the correlation between the phylogenetic, abundance and rank-based distances among pairs of OTUs. The computation of complete distance matrices was hampered by the large number of OTUs involved, e.g. 90 432 amounts to a distance matrix of 90 432^2 = 8.18 billions of cells. To circumvent this, we created 999 random subsets of 100 OTUs from which we created and compared the phylogenetic to the abundance and rank-based distance matrices (10 000 cells). The phylogenetic distance matrix was computed in R following [17], using OTUs V4 sequences and functions 'AlignSeqs()' and 'dist.ml()' from R packages 'DECIPHER' [18] and 'phangorn' [19]. Both rank and abundance distance matrices were computed using Euclidean distance [4]. The correlation between distance-matrices was studied using Pearson's correlation coefficient (R^2 and p.value) and a linear regression, the coefficients were collated for the 999 OTUs subsets.

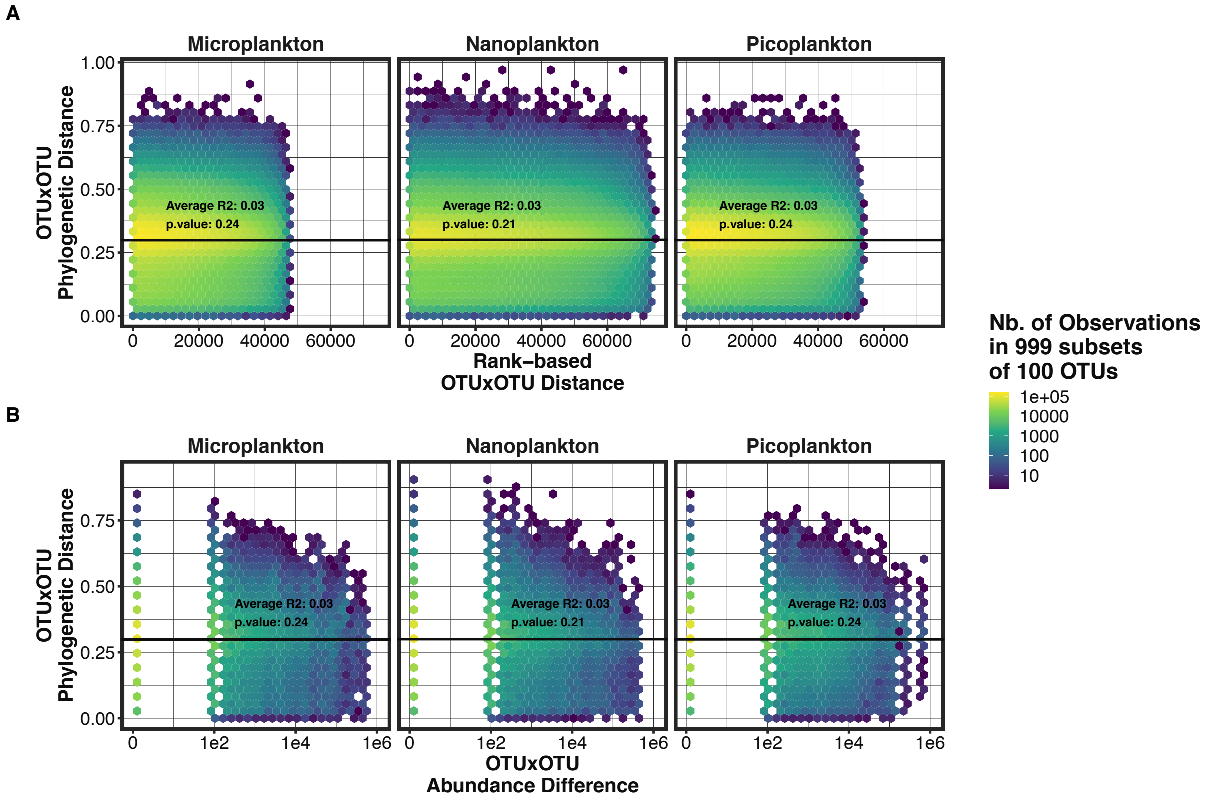

Figure S4: Testing the phylogenetic signal of rarity among protists. A) Relation between OTUs' phylogenetic distance and distance within the rank abundance curves of micro, nano and pico-plankton. These results originate from 999 subsets of 100 OTUs per size-fraction. Pearson correlation was performed and a linear model was fitted on the results of each subset. The linear model and the correlation on these plots correspond to the averaged coefficients across the subsamples. B) Relation between OTUs' phylogenetic distance and abundance distance (computed as the difference in number of reads in each respective size-fraction). This plot follows the same computational procedures.

In average, the correlation of phylogeny with rank and phylogeny with abundance were not significant (p.value > 0.05). This was confirmed with the linear models which did not show any

trend (Figure S4). We conclude that there exists no phylogenetic signal for rarity among marine protists, i.e. abundant or rare taxa are not composed of specific phyla.

Supplementary Material 6: Quantifying functional diversity in the rare protistan biosphere.

To compute metrics of functional diversity we used the subset of functionally annotated OTUs (41 614) and R package 'mFD'. Only non-weighted metrics were computed [20]. Functional richness represents the breadth of ecological strategies in a community (a high number of ecological strategies leads to high functional richness), functional dispersion represents how evenly distributed are the ecological strategies in this breadth (the higher the value the more a community is dominated by a specific ecological strategy) [21]. Functional metrics were computed by aggregating OTUs at the taxonomic reference level, i.e. we studied the presence/absence of 1380 protistan taxonomic references annotated with 13 traits. Not all subsets of rank abundance curves harbored the same amount of annotated taxonomic references, yielding between 315 and 583 taxonomic reference per subset.

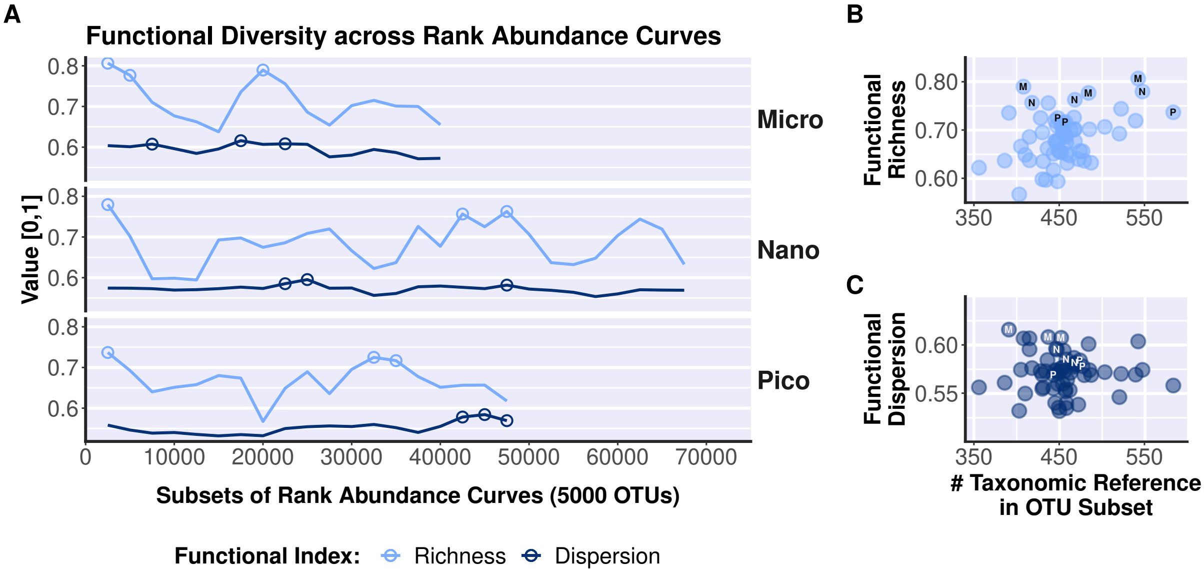

Figure S5: Functional diversity of marine protists along rank abundance curves (A) and relationship with the number of taxonomic references per subset (B). Functional richness represents the breadth of ecological strategies in a community, functional dispersion represents how evenly distributed are the ecological strategies in this breadth. Both indexes were computed for each subsets of rank abundance curves containing 5000 OTUs, using the taxonomic references annotated with traits (315 and 583 taxonomic references across all subsets) and R package 'mFD'. The 3 highest values of each proxy in each size-fraction were circled and highlighted by a letter corresponding to the size-fraction in the relationship plots ("M" for micro, "N" for nano and "P" for pico-plankton).

Along rank abundance curves, both functional richness (ranging between 0.57 and 0.81) and dispersion (ranging between 0.53 and 0.62) were high suggesting that ecological strategies were numerous but that subsets were often dominated by few similar strategies (Figure S5A). Both functional richness and dispersion were only weakly correlated to the number of taxonomic references annotated with traits per subsets (Figure S5B), suggesting that the values were not affected by the quality of the trait annotation among subsets. In general, both functional richness, 0.71, 0.68 and 0.67 respectively in micro, nano and pico-plankton, and dispersion, 0.59, 0.57 and 0.55, were lower in smaller size-fractions. High values of functional richness were observed both among subsets of abundant and rare OTUs, in all size-fractions

(Figure S5A). Functional dispersion showed less variability (Figures S5A). Nevertheless, dispersion decreased towards the rarest subsets in the micro-plankton but increased in those of the pico-plankton (Figure S5A), indicating that ecological strategies were more evenly distributed among rare micro-sized protists, and more dominated by few strategies among rare pico-sized protists.

Supplementary material 7: Analyses with OTUs clustered at 95% of identity.

Aware that the phylogenetic resolution of our study could affect our results [47,48], we generated a second dataset in which the Swarm2-OTUs were further clustered at 95% of identity using vsearch (--cluster_size 0.95) [49]. We reproduced: 1) the RACs per-size fraction and subsets of 5000 OTUs (see section *Defining rarity* in the Material and Methods of the original manuscript), 2) the computation of assembly processes and PERMANOVA (see section *Inferring the assembly processes…* in the Material and Methods of the original manuscript), and, 3) the analysis of the link between phylogeny and abundance (see section *Correlation between rarity, traits, and phylogeny* in the Material and Methods of the original manuscript).

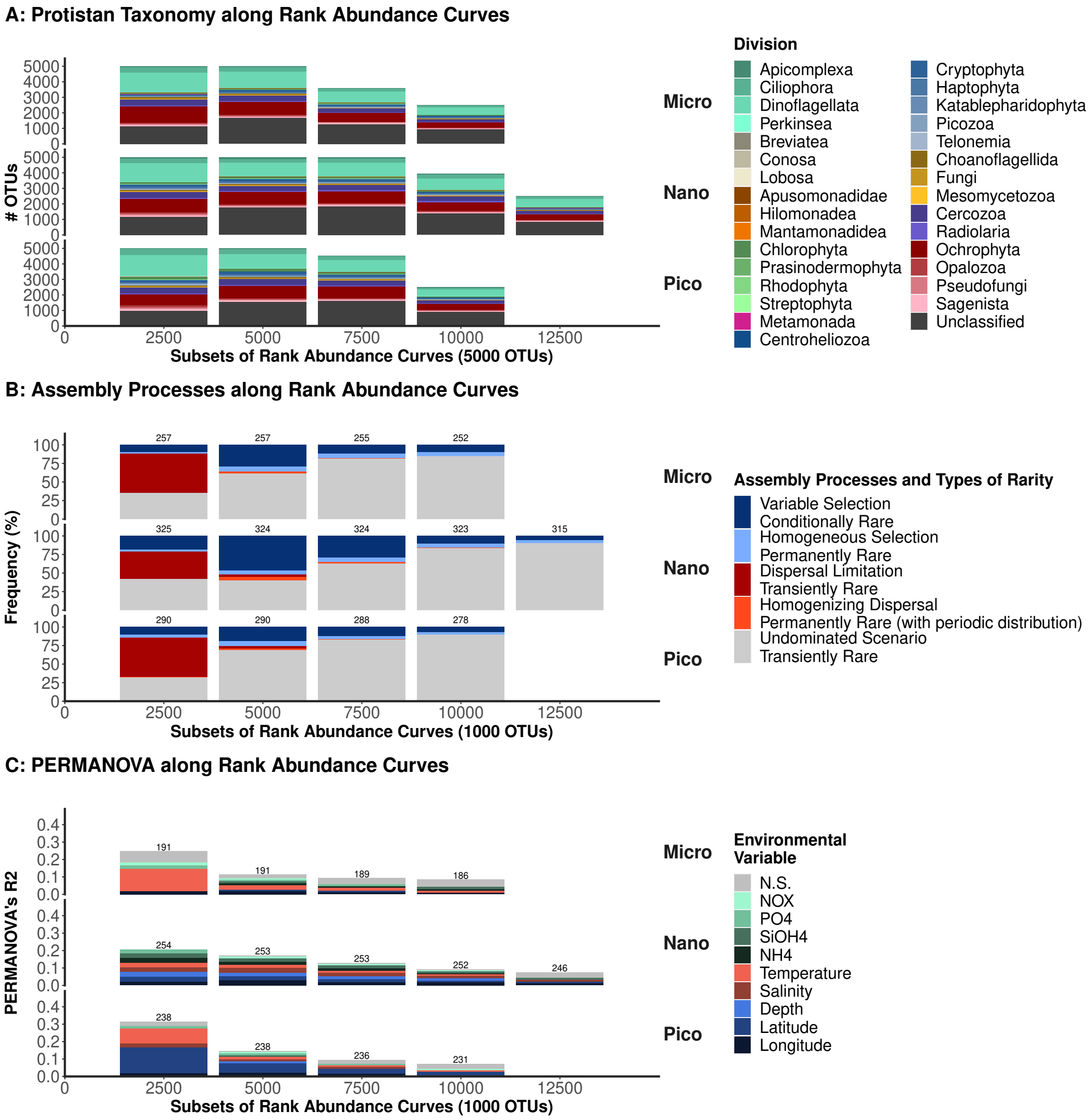

Figure S6: Protistan taxonomic composition, underlying assembly processes and environmental drivers along the rank abundance curves of our datasets with OTUs clustered at 95% of identity. Abundant OTUs (left side of all graphs) were selected using the multivariate approach of ref. [5] . Subsets of 5000 OTUs were computed with a sliding window along rank abundance curves (X axis on all plots). The last two subsets contain less OTUs because rank abundance curves are not exact multiples of 2500. A) Taxonomy is given at the Division level (as annotated with the PR2 database v4.13.0), taxonomic ranks were sorted by Supergroups. B) Assembly processes were inferred from the distribution of the OTUs in each subset using R package iCAMP ref. [6], the number of samples from which assembly processes were inferred was annotated on top of each barplot (the same procedure was applied in C). Assembly processes were converted into types of rarity following the nomenclature of ref. [3]. C) PERMANOVAs were computed using the ßMNTD of each subset (computed during the inference of assembly processes) and the environmental variables measured in our survey. The barplots represent the total R^2 of each PERMANOVA and the color code represent the contribution of each variable to the fit. The overall fit sometimes includes variables for which the individual fit is non-significant (see the gray color code, N.S. stands for not-significant), not to be confused with residual correlation (= 1 – overall fit).

With a lower number of OTUs (8589, 11441 and 9538 OTUs respectively for micro, nano and pico-plankton) and thus less subsets of 5000 OTUs along RACs, we retrieved generally similar results as the ones observed in Figure S3. Briefly, taxonomic diversity remained stable along RACs despite an increase in taxonomically unclassified OTUs towards the rarest-end of RACs, from 22 to 36%, 23 to 33%, and 19 to 36% in the most abundant subset to the rarest, respectively for micro, nano, and pico-plankton (Figure S6A). Assembly processes showed a dominance of *dispersal limitations* in the first subset containing the 5000 more abundant OTUs (53, 37, and 54% of assembly processes, respectively for micro, nano, and picoplankton), a peak in *variable selection* in intermediate levels of rarity (29, 46, and 19%, respectively for micro, nano, and pico-plankton) and a larger influence of stochastic processes (see the proportion of *undominated scenario*, 85, 90, and 89%, respectively for micro, nano, and pico-plankton) in the rare-end of the RACs (Figure S6B). *Homogenizing Selection* persisted in low proportions along the rare subsets of RACs (between 3 and 6%, Figure S6B). The same environmental variables fitted with the phylogenetic turnover of the different size fraction as in the results observed in Figure S3 (see supplementary material 4 for interpretation). We observed the same decrease in explicative power after the first subset of RACs (R^2 from 0.25 to 0.08, 0.20 to 0.08, and 0.31 to 0.07, respectively for the micro, nano, and pico-plankton).

We then studied whether the correlation between phylogeny, rank and abundance changed in the datatset with OTUs clustered at 95% of identity (Figure S7). The average p.value of the correlation between variables across subsets of 100 OTUs were non-significant except for the pico-plankton (average p.value of 0.4). Nevertheless, the average trends (displayed in the linear model) and distribution of the variables showed no co-variance between phylogeny, rank and abundance. We conclude that at this coarser phylogenetic resolution, there was also no phylogenetic signal for rarity among marine protists, i.e. abundant or rare taxa are not composed of specific phyla.

Overall, despite a clear effect on the number of OTUs included in each analysis, the results of supplementary material 7 showed that the phylogenetic resolution (percentage of identity at which OTUs are clustered) had little effect on the patterns we observed in the original

dataset (see manuscript). This, and the large sampling coverage of our study, suggest that the results and hypotheses presented in our manuscript could be extrapolated to other communities sampled at the regional scale.

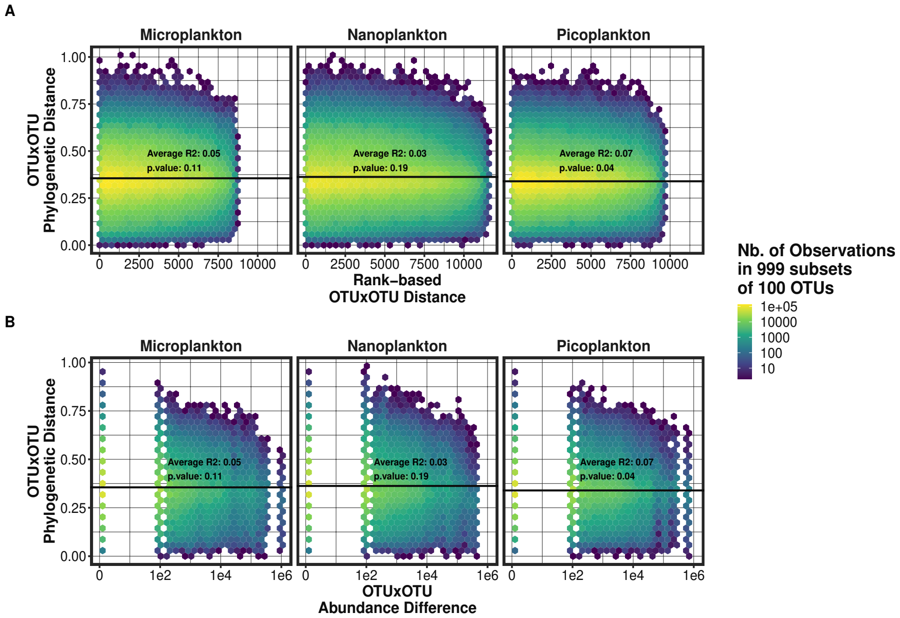

Figure S7: Testing the phylogenetic signal of rarity among protists based on the dataset of OTUs clustered at 95% of identity. A) Relation between OTUs' phylogenetic distance and distance within the rank abundance curves of micro, nano and pico-plankton. These results originate from 999 subsets of 100 OTUs per size-fraction. Pearson correlation was performed and a linear model was fitted on the results of each subset. The linear model and the correlation on these plots correspond to the averaged coefficients across the subsamples. B) Relation between OTUs' phylogenetic distance and abundance distance (computed as the difference in number of reads in each respective size-fraction). This plot follows the same computational procedures.

References

1. Hsieh TC, Ma KH, Chao A. iNEXT: an R package for rarefaction and extrapolation of species diversity (Hill numbers). Methods Ecol Evol. 2016;7:1451–6.

2. Sauvadet A-L, Gobet A, Guillou L. Comparative analysis between protist communities from the deep-sea pelagic ecosystem and specific deep hydrothermal habitats. Environ Microbiol. 2010;12:2946–64.

3. Jia X, Dini-Andreote F, Falcão Salles J. Community Assembly Processes of the Microbial Rare Biosphere. Trends Microbiol [Internet]. Elsevier Ltd; 2018;26:738–47. Available from: https://doi.org/10.1016/j.tim.2018.02.011

4. Legendre P, Legendre L. Numerical Ecology. Third English Edition. Third Engl. Developments in Environmental Modelling 24, Numerical, editors. Amsterdam (The Netherlands): Elsevier; 2012.

5. Gobet A, Quince C, Ramette A. Multivariate cutoff level analysis (MultiCoLA) of large community data sets. Nucleic Acids Res. 2010;38.

6. Ning D, Yuan M, Wu L, Zhang Y, Guo X, Zhou X, et al. A quantitative framework reveals ecological drivers of grassland microbial community assembly in response to warming. Nat Commun [Internet]. Springer US; 2020;11. Available from: http://dx.doi.org/10.1038/s41467-020-18560-z

7. Oksanen J, Blanchet FG, Friendly M, Kindt R, Legendre P, Mcglinn D, et al. vegan: Community Ecology Package [Internet]. 2018. Available from: https://cran.r-project.org/package=vegan

8. Ladau J, Eloe-Fadrosh EA. Spatial, Temporal, and Phylogenetic Scales of Microbial Ecology. Trends Microbiol [Internet]. The Author(s); 2019;27:662–9. Available from: https://doi.org/10.1016/j.tim.2019.03.003

9. Cloern JE. Phytoplankton bloom dynamics in coastal ecosystems: A review with some general lessons from sustained investigation of San Francisco Bay, California. Rev Geophys. 1996;34:127.

10. Litchman E, Klausmeier CA, Schofield OM, Falkowski PG. The role of functional traits and trade-offs in structuring phytoplankton communities: Scaling from cellular to ecosystem level. Ecol Lett. 2007;10:1170–81.

11. Ramond P, Sourisseau M, Simon N, Romac S, Schmitt S, Rigaut-Jalabert F, et al. Coupling between taxonomic and functional diversity in protistan coastal communities. Environ Microbiol [Internet]. John Wiley & Sons, Ltd (10.1111); 2019;21:730–49. Available from: https://doi.org/10.1111/1462-2920.14537

12. Dolan JR. Phosphorus and ammonia excretion by planktonic protists. Mar Geol. 1997;139:109–22.

13. Legendre L, Rassoulzadegan F. Plankton and nutrient dynamics in marine waters.

Ophelia. 1995;41:153–72.
14. Maguer JF, L'Helguen S, Caradec J, Klein C. Size-dependent uptake of nitrate and ammonium as a function of light in well-mixed temperate coastal waters. Cont Shelf Res. 2011;31:1620–31.
15. Burki F, Sandin MM, Jamy M. Diversity and ecology of protists revealed by metabarcoding. Curr Biol [Internet]. The Authors; 2021;31:R1267–80. Available from: https://doi.org/10.1016/j.cub.2021.07.066
16. Sommeria-Klein G, Watteaux R, Ibarbalz FM, Pierella Karlusich JJ, Iudicone D, Bowler C, et al. Global drivers of eukaryotic plankton biogeography in the sunlit ocean. Science (80-). 2021;374:594–9.
17. Callahan BJ, Sankaran K, Fukuyama JA, McMurdie PJ, Holmes SP. Bioconductor workflow for microbiome data analysis: From raw reads to community analyses [version 1; referees: 3 approved]. F1000Research. 2016;5:1–49.
18. Wright ES. Using DECIPHER v2.0 to analyze big biological sequence data in R. R J. 2016;8:352–9.
19. Schliep KP. phangorn: Phylogenetic analysis in R. Bioinformatics. 2011;27:592–3.
20. Bálint M, Bahram M, Eren AM, Faust K, Fuhrman JA, Lindahl B, et al. Millions of reads, thousands of taxa: microbial community structure and associations analyzed via marker genes. FEMS Microbiol Rev [Internet]. 2016;6:189–96. Available from: http://www.ncbi.nlm.nih.gov/pubmed/27358393
21. Mouillot D, Gaham NAJ, Villéger S, Mason NWH, Bellwood DR. A functional approach reveals community responses to disturbances. Trends Ecol Evol. 2013;28:167–77.

Supplementary Table 1: Information on the number of OTUs, number of taxonomic references and number of OTUs susccessfully annotated with traits across Eukaryotic Divisions.

**Taxonomy**		**OTUs and Taxonomic references**			**Trait annotation**	
Kingdom	Division	Number of OTUs	Number of unique taxonomic refererences	Average number of taxonomic reference per OTU	Number of OTUs with trait annotation	% of OTUs annotated with trait
Eukaryota	Alveolata	31170	640	48,70	15980	51,26724415
Eukaryota	Apusomonadida	83	21	3,95	83	100
Eukaryota	Breviatida	5	3	1,67	5	100
Eukaryota	Centrohelida	136	28	4,86	90	66,17647059
Eukaryota	Conosa	5	5	1,00	5	100
Eukaryota	Cryptophyta	2656	19	139,79	2651	99,81174699
Eukaryota	Haptophyta	1279	46	27,80	742	58,01407349
Eukaryota	Holomycota	706	105	6,72	395	55,9490085
Eukaryota	Holozoa	1342	37	36,27	997	74,29210134
Eukaryota	Katablepharidida	521	13	40,08	481	92,32245681
Eukaryota	Lobosa	47	14	3,36	47	100
Eukaryota	Mantamonadida	1	1	1,00	1	100
Eukaryota	Metamonada	7	3	2,33	7	100
Eukaryota	Microhelida	1	1	1,00	1	100
Eukaryota	Picomonadida	1278	2	639,00	1278	100
Eukaryota	Planomonadida	21	10	2,10	21	100
Eukaryota	Rhizaria	5556	211	26,33	1923	34,6112311
Eukaryota	Rigifilida	3	1	3,00	3	100
Eukaryota	Stramenopiles	22839	386	59,17	12666	55,4577696
Eukaryota	Telonemida	843	5	168,60	843	100
Eukaryota	Viridiplantae	3520	99	35,56	3395	96,44886364
Eukaryota	Unclassified	18413	30	613,77	0	0
Eukaryota	Total	90432	1680	53,83	41614	46,01689667

Supplementary Table 2: Total abundance (# of reads), occurence (# of samples), rank, and taxonomy of abundant OTUs across the three size fractions (micro, nano, and picoplankton).

otu ID	SF	Nb of Reads	Nb of Occurrence	Rank	Kingdom	Supergroup	Division	Class	Order	Family	Genus	Species
11	Micro	461349	256	1	Eukaryota	Alveolata	Dinoflagellata	Dinophyceae	Gymnodiniales	Gymnodiniaceae	Gyrodinium	Gyrodinium_spirale
19	Micro	326629	211	2	Eukaryota	Alveolata	Dinoflagellata	Dinophyceae	Gonyaulacales	Ceratiaceae	Tripos	Tripos_fusus
5	Micro	234638	257	3	Eukaryota	Alveolata	Dinoflagellata	Dinophyceae	Gymnodiniales	Kareniaceae	Karenia	Karenia_brevis
28	Micro	172879	167	4	Eukaryota	Alveolata	Unclassified	Unclassified	Unclassified	Unclassified	Unclassified	Unclassified
39	Micro	145038	168	5	Eukaryota	Alveolata	Dinoflagellata	Dinophyceae	Unclassified	Unclassified	Unclassified	Unclassified
20	Micro	140373	224	6	Eukaryota	Stramenopiles	Ochrophyta	Bacillariophyta	Bacillariophyta_X	Radial-centric-basal-Coscinodiscophyceae	Guinardia	Guinardia_delicatula
4	Micro	140000	256	7	Eukaryota	Alveolata	Dinoflagellata	Dinophyceae	Peridiniales	Heterocapsaceae	Heterocapsa	Unclassified
44	Micro	121482	115	8	Eukaryota	Alveolata	Dinoflagellata	Dinophyceae	Gymnodiniales	Warnowiaceae	Warnowia	Warnowia_sp.
46	Micro	121252	144	9	Eukaryota	Alveolata	Dinoflagellata	Dinophyceae	Gymnodiniales	Gymnodiniaceae	Unclassified	Unclassified
26	Micro	112429	193	10	Eukaryota	Rhizaria	Radiolaria	Acantharea	Chaunacanthida	Chaunacanthida_X	Acanthometron	Acanthometron_sp.
62	Micro	109559	119	11	Eukaryota	Alveolata	Dinoflagellata	Dinophyceae	Gonyaulacales	Goniodomataceae	Alexandrium	Unclassified
42	Micro	105768	170	12	Eukaryota	Alveolata	Dinoflagellata	Dinophyceae	Gymnodiniales	Gymnodiniaceae	Gyrodinium	Gyrodinium_heterogrammum
31	Micro	98613	200	13	Eukaryota	Alveolata	Dinoflagellata	Dinophyceae	Gymnodiniales	Gymnodiniaceae	Lepidodinium	Lepidodinium_chlorophorum
33	Micro	94753	172	14	Eukaryota	Stramenopiles	Ochrophyta	Bacillariophyta	Bacillariophyta_X	Radial-centric-basal-Coscinodiscophyceae	Leptocylindrus	Leptocylindrus_convexus
38	Micro	86742	242	15	Eukaryota	Alveolata	Dinoflagellata	Syndiniales	Dino-Group-I	Dino-Group-I-Clade-1	Dino-Group-I-Clade-1_X	Dino-Group-I-Clade-1_X_sp.
78	Micro	83312	140	16	Eukaryota	Alveolata	Dinoflagellata	Noctilucophyceae	Noctilucales	Noctilucaceae	Noctiluca	Noctiluca_scintillans
7	Micro	81514	244	17	Eukaryota	Stramenopiles	Ochrophyta	Bacillariophyta	Bacillariophyta_X	Polar-centric-Mediophyceae	Chaetoceros	Chaetoceros_tenuissimus
18	Micro	80566	225	18	Eukaryota	Alveolata	Dinoflagellata	Dinophyceae	Gymnodiniales	Unclassified	Unclassified	Unclassified
8	Micro	77226	213	19	Eukaryota	Stramenopiles	Ochrophyta	Bacillariophyta	Bacillariophyta_X	Polar-centric-Mediophyceae	Minidiscus	Minidiscus_comicus
1	Micro	75629	180	20	Eukaryota	Alveolata	Dinoflagellata	Dinophyceae	Gonyaulacales	Goniodomataceae	Alexandrium	Unclassified
54	Micro	73403	112	21	Eukaryota	Rhizaria	Cercozoa	Filosa-Thecofilosea	Ebriida	Ebriidae	Ebria	Ebria_tripartita
23	Micro	73388	252	22	Eukaryota	Stramenopiles	Ochrophyta	Bacillariophyta	Bacillariophyta_X	Polar-centric-Mediophyceae	Minidiscus	Minidiscus_variabilis
40	Micro	69602	58	23	Eukaryota	Alveolata	Dinoflagellata	Dinophyceae	Gymnodiniales	Warnowiaceae	Warnowia	Warnowia_sp.
60	Micro	65914	138	24	Eukaryota	Stramenopiles	Opalozoa	MAST-12	Unclassified	Unclassified	Unclassified	Unclassified
72	Micro	58532	64	25	Eukaryota	Alveolata	Dinoflagellata	Dinophyceae	Unclassified	Unclassified	Unclassified	Unclassified
53	Micro	51459	105	26	Eukaryota	Alveolata	Dinoflagellata	Dinophyceae	Prorocentrales	Prorocentraceae	Prorocentrum	Unclassified
80	Micro	49969	104	27	Eukaryota	Stramenopiles	Ochrophyta	Bacillariophyta	Bacillariophyta_X	Polar-centric-Mediophyceae	Unclassified	Unclassified
123	Micro	44354	112	28	Eukaryota	Alveolata	Dinoflagellata	Dinophyceae	Gymnodiniales	Ceratoperidiniaceae	Ceratoperidinium	Ceratoperidinium_falcatum
68	Micro	44251	74	29	Eukaryota	Alveolata	Dinoflagellata	Dinophyceae	Gymnodiniales	Unclassified	Unclassified	Unclassified
116	Micro	42734	117	30	Eukaryota	Alveolata	Dinoflagellata	Dinophyceae	Gymnodiniales	Chytriodiniaceae	Unclassified	Unclassified
41	Micro	40509	195	31	Eukaryota	Stramenopiles	Ochrophyta	Bacillariophyta	Bacillariophyta_X	Polar-centric-Mediophyceae	Unclassified	Unclassified
51	Micro	40280	240	32	Eukaryota	Alveolata	Dinoflagellata	Dinophyceae	Suessiales	Suessiaceae	Biecheleria	Biecheleria_sp.
169	Micro	37300	11	33	Eukaryota	Rhizaria	Radiolaria	Polycystinea	Collodaria	Sphaerozoidae	Collozoum	Collozoum_inerme
25	Micro	34610	229	34	Eukaryota	Alveolata	Dinoflagellata	Dinophyceae	Gymnodiniales	Gymnodiniaceae	Gyrodinium	Gyrodinium_helveticum
63	Micro	31532	206	35	Eukaryota	Stramenopiles	Stramenopiles_X	Stramenopiles_X-Group-7	Stramenopiles_X-Group-7_X	Stramenopiles_X-Group-7_XX	Stramenopiles_X-Group-7_XXX	Stramenopiles_X-Group-7_XXX_sp.
29	Micro	29845	88	36	Eukaryota	Alveolata	Dinoflagellata	Dinophyceae	Gonyaulacales	Gonyaulacaceae	Gonyaulax	Gonyaulax_spinifera
95	Micro	27186	126	37	Eukaryota	Stramenopiles	Ochrophyta	Bacillariophyta	Bacillariophyta_X	Raphid-pennate	Pseudo-nitzschia	Unclassified
64	Micro	26609	211	38	Eukaryota	Alveolata	Ciliophora	Spirotrichea	Choreotrichida	Strobilidiidae_I	Pelagostrobilidium	Pelagostrobilidium_neptuni
5	Nano	420419	325	1	Eukaryota	Alveolata	Dinoflagellata	Dinophyceae	Gymnodiniales	Kareniaceae	Karenia	Karenia_brevis
11	Nano	407695	325	2	Eukaryota	Alveolata	Dinoflagellata	Dinophyceae	Gymnodiniales	Gymnodiniaceae	Gyrodinium	Gyrodinium_spirale
4	Nano	388757	325	3	Eukaryota	Alveolata	Dinoflagellata	Dinophyceae	Peridiniales	Heterocapsaceae	Heterocapsa	Unclassified
18	Nano	247007	312	4	Eukaryota	Alveolata	Dinoflagellata	Dinophyceae	Gymnodiniales	Unclassified	Unclassified	Unclassified
10	Nano	211664	310	5	Eukaryota	Alveolata	Dinoflagellata	Dinophyceae	Unclassified	Unclassified	Unclassified	Unclassified
8	Nano	202808	292	6	Eukaryota	Stramenopiles	Ochrophyta	Bacillariophyta	Bacillariophyta_X	Polar-centric-Mediophyceae	Minidiscus	Minidiscus_comicus
25	Nano	155701	318	7	Eukaryota	Alveolata	Dinoflagellata	Dinophyceae	Gymnodiniales	Gymnodiniaceae	Gyrodinium	Gyrodinium_helveticum
23	Nano	150802	321	8	Eukaryota	Stramenopiles	Ochrophyta	Bacillariophyta	Bacillariophyta_X	Polar-centric-Mediophyceae	Minidiscus	Minidiscus_variabilis
22	Nano	119674	321	9	Eukaryota	Hacrobia	Cryptophyta	Cryptophyceae	Cryptomonadales	Cryptomonadales_X	Plagioselmis	Plagioselmis_prolonga
32	Nano	113909	325	10	Eukaryota	Alveolata	Dinoflagellata	Dinophyceae	Gymnodiniales	Gymnodiniaceae	Gymnodinium	Gymnodinium_sp.
16	Nano	107617	268	11	Eukaryota	Hacrobia	Cryptophyta	Cryptophyceae	Cryptomonadales	Cryptomonadales_X	Teleaulax	Teleaulax_acuta
2	Nano	107485	306	12	Eukaryota	Archaeplastida	Chlorophyta	Mamiellophyceae	Mamiellales	Bathycoccaceae	Ostreococcus	Ostreococcus_lucimarinus
30	Nano	105866	319	13	Eukaryota	Hacrobia	Cryptophyta	Cryptophyceae	Cryptomonadales	Cryptomonadales_X	Teleaulax	Teleaulax_gracilis
28	Nano	105161	240	14	Eukaryota	Alveolata	Unclassified	Unclassified	Unclassified	Unclassified	Unclassified	Unclassified
20	Nano	100827	296	15	Eukaryota	Stramenopiles	Ochrophyta	Bacillariophyta	Bacillariophyta_X	Radial-centric-basal-Coscinodiscophyceae	Guinardia	Guinardia_delicatula
38	Nano	94441	292	16	Eukaryota	Alveolata	Dinoflagellata	Syndiniales	Dino-Group-I	Dino-Group-I-Clade-1	Dino-Group-I-Clade-1_X	Dino-Group-I-Clade-1_X_sp.
40	Nano	88662	53	17	Eukaryota	Alveolata	Dinoflagellata	Dinophyceae	Gymnodiniales	Warnowiaceae	Warnowia	Warnowia_sp.
33	Nano	88556	181	18	Eukaryota	Stramenopiles	Ochrophyta	Bacillariophyta	Bacillariophyta_X	Radial-centric-basal-Coscinodiscophyceae	Leptocylindrus	Leptocylindrus_convexus
31	Nano	84194	284	19	Eukaryota	Alveolata	Dinoflagellata	Dinophyceae	Gymnodiniales	Gymnodiniaceae	Lepidodinium	Lepidodinium_chlorophorum
7	Nano	84137	311	20	Eukaryota	Stramenopiles	Ochrophyta	Bacillariophyta	Bacillariophyta_X	Polar-centric-Mediophyceae	Chaetoceros	Chaetoceros_tenuissimus
48	Nano	77518	322	21	Eukaryota	Alveolata	Dinoflagellata	Dinophyceae	Unclassified	Unclassified	Unclassified	Unclassified
41	Nano	75200	284	22	Eukaryota	Stramenopiles	Ochrophyta	Bacillariophyta	Bacillariophyta_X	Polar-centric-Mediophyceae	Unclassified	Unclassified
56	Nano	73226	305	23	Eukaryota	Alveolata	Dinoflagellata	Dinophyceae	Peridiniales	Heterocapsaceae	Heterocapsa	Heterocapsa_nei/rotundata
93	Nano	66758	319	24	Eukaryota	Alveolata	Dinoflagellata	Dinophyceae	Gymnodiniales	Gymnodiniaceae	Gyrodinium	Gyrodinium_dominans
74	Nano	62704	221	25	Eukaryota	Unclassified	Unclassified	Unclassified	Unclassified	Unclassified	Unclassified	Unclassified
60	Nano	61721	144	26	Eukaryota	Stramenopiles	Opalozoa	MAST-12	Unclassified	Unclassified	Unclassified	Unclassified
39	Nano	58289	202	27	Eukaryota	Alveolata	Dinoflagellata	Dinophyceae	Unclassified	Unclassified	Unclassified	Unclassified
26	Nano	57541	201	28	Eukaryota	Rhizaria	Radiolaria	Acantharea	Chaunacanthida	Chaunacanthida_X	Acanthometron	Acanthometron_sp.
59	Nano	56386	302	29	Eukaryota	Hacrobia	Cryptophyta	Cryptophyceae	Cryptomonadales	Cryptomonadales_X	Unclassified	Unclassified
45	Nano	56093	273	30	Eukaryota	Stramenopiles	Ochrophyta	Bacillariophyta	Bacillariophyta_X	Radial-centric-basal-Coscinodiscophyceae	Leptocylindrus	Leptocylindrus_sp.
42	Nano	54745	225	31	Eukaryota	Alveolata	Dinoflagellata	Dinophyceae	Gymnodiniales	Gymnodiniaceae	Gyrodinium	Gyrodinium_heterogrammum
78	Nano	51471	160	32	Eukaryota	Alveolata	Dinoflagellata	Noctilucophyceae	Noctilucales	Noctilucaceae	Noctiluca	Noctiluca_scintillans
2	Pico	628227	290	1	Eukaryota	Archaeplastida	Chlorophyta	Mamiellophyceae	Mamiellales	Bathycoccaceae	Ostreococcus	Ostreococcus_lucimarinus
15	Pico	244477	290	2	Eukaryota	Archaeplastida	Chlorophyta	Mamiellophyceae	Mamiellales	Mamiellaceae	Micromonas	Micromonas_commoda_A2
21	Pico	222671	289	3	Eukaryota	Archaeplastida	Chlorophyta	Mamiellophyceae	Mamiellales	Bathycoccaceae	Bathycoccus	Bathycoccus_prasinos
4	Pico	128778	289	4	Eukaryota	Alveolata	Dinoflagellata	Dinophyceae	Peridiniales	Heterocapsaceae	Heterocapsa	Unclassified
14	Pico	128185	286	5	Eukaryota	Archaeplastida	Chlorophyta	Mamiellophyceae	Mamiellales	Mamiellaceae	Micromonas	Micromonas_bravo_B2
35	Pico	127188	290	6	Eukaryota	Archaeplastida	Chlorophyta	Mamiellophyceae	Mamiellales	Mamiellaceae	Micromonas	Unclassified
5	Pico	117091	290	7	Eukaryota	Alveolata	Dinoflagellata	Dinophyceae	Gymnodiniales	Kareniaceae	Karenia	Karenia_brevis
22	Pico	112722	289	8	Eukaryota	Hacrobia	Cryptophyta	Cryptophyceae	Cryptomonadales	Cryptomonadales_X	Plagioselmis	Plagioselmis_prolonga
10	Pico	110219	277	9	Eukaryota	Alveolata	Dinoflagellata	Dinophyceae	Unclassified	Unclassified	Unclassified	Unclassified
25	Pico	92313	269	10	Eukaryota	Alveolata	Dinoflagellata	Dinophyceae	Gymnodiniales	Gymnodiniaceae	Gyrodinium	Gyrodinium_helveticum
30	Pico	87273	288	11	Eukaryota	Hacrobia	Cryptophyta	Cryptophyceae	Cryptomonadales	Cryptomonadales_X	Teleaulax	Teleaulax_gracilis
43	Pico	82190	281	12	Eukaryota	Hacrobia	Picozoa	Picozoa_X	Picozoa_XX	Picozoa_XXX	Picozoa_XXXX	Picozoa_XXXX_sp.
11	Pico	79986	289	13	Eukaryota	Alveolata	Dinoflagellata	Dinophyceae	Gymnodiniales	Gymnodiniaceae	Gyrodinium	Gyrodinium_spirale
38	Pico	72639	266	14	Eukaryota	Alveolata	Dinoflagellata	Syndiniales	Dino-Group-I	Dino-Group-I-Clade-1	Dino-Group-I-Clade-1_X	Dino-Group-I-Clade-1_X_sp.
32	Pico	64853	282	15	Eukaryota	Alveolata	Dinoflagellata	Dinophyceae	Gymnodiniales	Gymnodiniaceae	Gymnodinium	Gymnodinium_sp.
49	Pico	62024	250	16	Eukaryota	Alveolata	Dinoflagellata	Syndiniales	Dino-Group-I	Dino-Group-I-Clade-1	Dino-Group-I-Clade-1_X	Dino-Group-I-Clade-1_X_sp.
26	Pico	55277	176	17	Eukaryota	Rhizaria	Radiolaria	Acantharea	Chaunacanthida	Chaunacanthida_X	Acanthometron	Acanthometron_sp.
16	Pico	51758	219	18	Eukaryota	Hacrobia	Cryptophyta	Cryptophyceae	Cryptomonadales	Cryptomonadales_X	Teleaulax	Teleaulax_acuta
90	Pico	48449	194	19	Eukaryota	Alveolata	Dinoflagellata	Syndiniales	Dino-Group-I	Dino-Group-I-Clade-1	Dino-Group-I-Clade-1_X	Dino-Group-I-Clade-1_X_sp.
99	Pico	43871	103	20	Eukaryota	Alveolata	Dinoflagellata	Syndiniales	Dino-Group-II	Dino-Group-II-Clade-10-and-11	Dino-Group-II-Clade-10-and-11_X	Dino-Group-II-Clade-10-and-11_X_sp.
127	Pico	41545	133	21	Eukaryota	Stramenopiles	Ochrophyta	Bacillariophyta	Bacillariophyta_X	Polar-centric-Mediophyceae	Unclassified	Unclassified
107	Pico	40999	227	22	Eukaryota	Alveolata	Dinoflagellata	Syndiniales	Dino-Group-II	Dino-Group-II-Clade-20	Dino-Group-II-Clade-20_X	Dino-Group-II-Clade-20_X_sp.
117	Pico	40438	101	23	Eukaryota	Alveolata	Dinoflagellata	Syndiniales	Dino-Group-II	Dino-Group-II-Clade-7	Dino-Group-II-Clade-7_X	Dino-Group-II-Clade-7_X_sp.
148	Pico	40165	284	24	Eukaryota	Alveolata	Dinoflagellata	Dinophyceae	Unclassified	Unclassified	Unclassified	Unclassified
136	Pico	40094	33	25	Eukaryota	Alveolata	Dinoflagellata	Syndiniales	Dino-Group-II	Dino-Group-II-Clade-10-and-11	Dino-Group-II-Clade-10-and-11_X	Dino-Group-II-Clade-10-and-11_X_sp.
18	Pico	38449	271	26	Eukaryota	Alveolata	Dinoflagellata	Dinophyceae	Gymnodiniales	Unclassified	Unclassified	Unclassified
100	Pico	38293	282	27	Eukaryota	Stramenopiles	Sagenista	MAST-7	MAST-7B	MAST-7B_X	MAST-7B_XX	MAST-7B_XX_sp.
147	Pico	37870	249	28	Eukaryota	Alveolata	Ciliophora	Spirotrichea	Strombidiida	Strombidiidae_M	Unclassified	Unclassified
94	Pico	37457	167	29	Eukaryota	Alveolata	Ciliophora	Spirotrichea	Strombidiida	Tontoniidae_A	Laboea	Unclassified
59	Pico	37031	264	30	Eukaryota	Hacrobia	Cryptophyta	Cryptophyceae	Cryptomonadales	Cryptomonadales_X	Unclassified	Unclassified
106	Pico	35748	255	31	Eukaryota	Alveolata	Ciliophora	Spirotrichea	Strombidiida_G	Strombidiida_G_X	Strombidiida_G_XX	Strombidiida_G_XX_sp.
98	Pico	34796	154	32	Eukaryota	Hacrobia	Picozoa	Picozoa_X	Picozoa_XX	Picozoa_XXX	Unclassified	Unclassified
48	Pico	34594	278	33	Eukaryota	Alveolata	Dinoflagellata	Dinophyceae	Unclassified	Unclassified	Unclassified	Unclassified
55	Pico	34525	243	34	Eukaryota	Archaeplastida	Chlorophyta	Mamiellophyceae	Mamiellales	Bathycoccaceae	Ostreococcus	Ostreococcus_tauri
8	Pico	34483	244	35	Eukaryota	Stramenopiles	Ochrophyta	Bacillariophyta	Bacillariophyta_X	Polar-centric-Mediophyceae	Minidiscus	Minidiscus_comicus
113	Pico	34479	239	36	Eukaryota	Alveolata	Dinoflagellata	Dinophyceae	Unclassified	Unclassified	Unclassified	Unclassified
83	Pico	33143	133	37	Eukaryota	Stramenopiles	Pseudofungi	MAST-1	MAST-1A	MAST-1A_X	MAST-1A_XX	MAST-1A_XX_sp.
66	Pico	32826	227	38	Eukaryota	Archaeplastida	Chlorophyta	Prasino-Clade-V	Pseudoscourfieldiales	Pycnococcaceae	Pycnococcaceae_X	Pycnococcaceae_X_sp.
60	Pico	32611	123	39	Eukaryota	Stramenopiles	Opalozoa	MAST-12	Unclassified	Unclassified	Unclassified	Unclassified
105	Pico	32314	123	40	Eukaryota	Alveolata	Dinoflagellata	Syndiniales	Dino-Group-II	Dino-Group-II-Clade-10-and-11	Dino-Group-II-Clade-10-and-11_X	Dino-Group-II-Clade-10-and-11_X_sp.

Supplementary Table 3: Name, ranks, number of OTUs, and number of OTUs with successfull trait annotation along the susbsets of 5000 OTUs of the rank abundance curves of micro, nano, and picoplankton.

Size Fraction	Subset	Fraction of the rank abundance curve	# of OTUs	# of OTUs annotated with traits	# of non-annotated OTUs	% annotated OTUs
Micro	Abundant	0-38	38	17	21	44,74
Micro	2500	0-5000	5000	2333	2667	46,66
Micro	5000	2500-7500	5000	2246	2754	44,92
Micro	7500	5000-10000	5000	2172	2828	43,44
Micro	10000	7500-12500	5000	2139	2861	42,78
Micro	12500	10000-15000	5000	2153	2847	43,06
Micro	15000	12500-17500	5000	2122	2878	42,44
Micro	17500	15000-20000	5000	2210	2790	44,20
Micro	20000	17500-22500	5000	2230	2770	44,60
Micro	22500	20000-25000	5000	2095	2905	41,90
Micro	25000	22500-27500	5000	2124	2876	42,48
Micro	27500	25000-30000	5000	2174	2826	43,48
Micro	30000	27500-32500	5000	2158	2842	43,16
Micro	32500	30000-35000	5000	2113	2887	42,26
Micro	35000	32500-37500	5000	2204	2796	44,08
Micro	37500	35000-40000	5000	2242	2758	44,84
Micro	40000	37500-42500	5000	2189	2811	43,78
Micro	42500	40000-44 813	4813	2098	2715	43,59
Micro	44813	42 313-44 813	2500	1080	1420	43,20
Nano	Abundant	0-32	32	12	20	37,50
Nano	2500	0-5000	5000	2317	2683	46,34
Nano	5000	2500-7500	5000	2169	2831	43,38
Nano	7500	5000-10000	5000	2076	2924	41,52
Nano	10000	7500-12500	5000	1995	3005	39,90
Nano	12500	10000-15000	5000	2031	2969	40,62
Nano	15000	12500-17500	5000	2054	2946	41,08
Nano	17500	15000-20000	5000	2013	2987	40,26
Nano	20000	17500-22500	5000	2021	2979	40,42
Nano	22500	20000-25000	5000	2110	2890	42,20
Nano	25000	22500-27500	5000	2139	2861	42,78
Nano	27500	25000-30000	5000	2160	2840	43,20
Nano	30000	27500-32500	5000	2103	2897	42,06
Nano	32500	30000-35000	5000	1980	3020	39,60
Nano	35000	32500-37500	5000	1964	3036	39,28
Nano	37500	35000-40000	5000	2175	2825	43,50
Nano	40000	37500-42500	5000	2233	2767	44,66
Nano	42500	40000-45000	5000	2042	2958	40,84
Nano	45000	42500-47500	5000	2088	2912	41,76
Nano	47500	45000-50000	5000	2282	2718	45,64
Nano	50000	47500-52500	5000	2451	2549	49,02
Nano	52500	50000-55000	5000	2372	2628	47,44
Nano	55000	52500-57500	5000	2236	2764	44,72
Nano	57500	55000-60000	5000	2264	2736	45,28
Nano	60000	57500-62500	5000	2270	2730	45,40
Nano	62500	60000-65000	5000	2469	2531	49,38
Nano	65000	62500-67500	5000	2584	2416	51,68
Nano	67500	65000-70000	5000	2517	2483	50,34
Nano	70000	67500-71380	3880	1965	1915	50,64
Nano	71380	68880-71380	2500	1264	1236	50,56
Pico_AB	Abundant	0-40	40	20	20	44,74
Pico_2500	2500	0-5000	5000	2656	2344	46,66
Pico_5000	5000	2500-7500	5000	2599	2401	44,92
Pico_7500	7500	5000-10000	5000	2576	2424	43,44
Pico_10000	10000	7500-12500	5000	2549	2451	42,78
Pico_12500	12500	10000-15000	5000	2515	2485	43,06
Pico_15000	15000	12500-17500	5000	2493	2507	42,44
Pico_17500	17500	15000-20000	5000	2538	2462	44,20
Pico_20000	20000	17500-22500	5000	2633	2367	44,60
Pico_22500	22500	20000-25000	5000	2531	2469	41,90
Pico_25000	25000	22500-27500	5000	2475	2525	42,48
Pico_27500	27500	25000-30000	5000	2539	2461	43,48
Pico_30000	30000	27500-32500	5000	2418	2582	43,16
Pico_32500	32500	30000-35000	5000	2342	2658	42,26
Pico_35000	35000	32500-37500	5000	2417	2583	44,08
Pico_37500	37500	35000-40000	5000	2438	2562	44,84
Pico_40000	40000	37500-42500	5000	2400	2600	43,78
Pico_42500	42500	40000-45000	5000	2308	2692	43,59
Pico_45000	45000	42500-47500	5000	2221	2779	43,20
Pico_47500	47500	45000-50000	5000	2277	2723	43,20
Pico_50000	50000	47500-51568	4068	1929	2139	43,20
Pico_52500	51568	49068-51568	2500	1198	1302	43,20
